# Comparative Genomics Determines Strain-Dependent Secondary Metabolite Production in *Streptomyces venezuelae* Strains

**DOI:** 10.3390/biom10060864

**Published:** 2020-06-05

**Authors:** Woori Kim, Namil Lee, Soonkyu Hwang, Yongjae Lee, Jihun Kim, Suhyung Cho, Bernhard Palsson, Byung-Kwan Cho

**Affiliations:** 1 Department of Biological Sciences and KI for the BioCentury, Korea Advanced Institute of Science and Technology, Daejeon 34141, Korea; alfodnfl@kaist.ac.kr (W.K.); pig948@kaist.ac.kr (N.L.); geager@kaist.ac.kr (S.H.); ahrk7247@kaist.ac.kr (Y.L.); kjhwlgns0997@kaist.ac.kr (J.K.); shcho95@kaist.ac.kr (S.C.); 2 Department of Bioengineering, University of California San Diego, La Jolla, CA 92093, USA; palsson@ucsd.edu; 3 Department of Pediatrics, University of California San Diego, La Jolla, CA 92093, USA; 4 Novo Nordisk Foundation Center for Biosustainability, Technical University of Denmark, 2800 Lyngby, Denmark; 5 Intelligent Synthetic Biology Center, Daejeon 34141, Korea

**Keywords:** comparative genomics, *Streptomyces venezuelae*, chloramphenicol, jadomycin, biosynthetic gene cluster

## Abstract

*Streptomyces venezuelae* is well known to produce various secondary metabolites, including chloramphenicol, jadomycin, and pikromycin. Although many strains have been classified as *S. venezuelae* species, only a limited number of strains have been explored extensively for their genomic contents. Moreover, genomic differences and diversity in secondary metabolite production between the strains have never been compared. Here, we report complete genome sequences of three *S. venezuelae* strains (ATCC 10712, ATCC 10595, and ATCC 21113) harboring chloramphenicol and jadomycin biosynthetic gene clusters (BGC). With these high-quality genome sequences, we revealed that the three strains share more than 85% of total genes and most of the secondary metabolite biosynthetic gene clusters (smBGC). Despite such conservation, the strains produced different amounts of chloramphenicol and jadomycin, indicating differential regulation of secondary metabolite production at the strain level. Interestingly, antagonistic production of chloramphenicol and jadomycin was observed in these strains. Through comparison of the chloramphenicol and jadomycin BGCs among the three strains, we found sequence variations in many genes, the non-coding RNA coding regions, and binding sites of regulators, which affect the production of the secondary metabolites. We anticipate that these genome sequences of closely related strains would serve as useful resources for understanding the complex secondary metabolism and for designing an optimal production process using *Streptomyces* strains.

## 1. Introduction

GC-rich genomes of Gram-positive bacteria *Streptomyces* encode a wide array of secondary metabolite biosynthetic gene clusters (smBGC) to produce various molecules of pharmaceutical and industrial importance such as antibiotics, anticancer drugs, antifungal agents, and herbicides [1,2,3]. Of those, *Streptomyces venezuelae* is characterized by the production of several secondary metabolites, including chloramphenicol, jadomycin, and pikromycin. The species is also regarded as an emerging model due to its fast growth rate, ease of genetic manipulation, lack of mycelial clumping, and sporulation in liquid media [4,5]. To the best of our knowledge, a total of 29 *S. venezuelae* strains have been identified (Table S1), and three of those (*S. venezuelae* ATCC 10712, NRRL B-65442, and ATCC 15439) have been widely used for the biosynthesis of secondary metabolites. First, *S. venezuelae* type strain ATCC 10712 produces chloramphenicol, which has a broad spectrum of antibiotic activity for the treatment of conjunctivitis, meningitis, and typhoid fever [6,7]. Additionally, under stress conditions, it produces jadomycin [8], which is an atypical angucycline antibiotic having antimicrobial and cytotoxic activity against multidrug-resistant breast cancer cell lines [9,10]. Many secondary metabolites, including venemycin [11], watasemycin [12], isodauc-8-en-11-ol [13], venezuelin [14], gaburedins [15], and foroxymithine [16], were also discovered in this strain. Second, NRRL B-65442 is known to produce chloramphenicol [17]. Lastly, the major secondary metabolites of ATCC 15439 are pikromycin derivatives due to its unusual ability to produce diverse macrolides from a single smBGC [18]. As it has a fast growth rate, relative ease of genetic manipulation, and an abundant precursor pool, ATCC 15439 has also been used as a heterologous expression host for the biosynthesis of secondary metabolites obtained from other organisms [19].

The recent genome mining approaches using high-quality genome sequences have shown the biosynthetic potential of *Streptomyces* to produce diverse secondary metabolites [20,21]. Interestingly, smBGCs are diverse at strain- as well as species-level [22,23]. For example, mining of 1100 *Streptomyces* genomes revealed that smBGCs are highly divergent even among closely related strains [23], which indicates the importance of exploring smBGCs at the strain-level. Moreover, comparative studies of *S. venezuelae* strains have not been explored to understand the differences between their genomes and secondary metabolism. In the present study, we completed genome sequences of three *S. venezuelae* strains (ATCC 10712, ATCC 10595, and ATCC 21113) harboring chloramphenicol biosynthetic gene cluster (BGC) to compare the conserved smBGCs in these strains and their genomic divergence. Importantly, since the previously reported genome sequence of ATCC 10712 contains thousands of undetermined sequences, including 9150 ‘N (any nucleotide)’s, 2 ‘M (A or C)’s, 2 ‘S (C or G)’s, and ‘K (G or T)’, we revisited the genetic information of the strain by determining a high-quality genome sequence. By comparing the completed genomes, we found genomic differences such as mutations in many genes, the non-coding RNA coding regions, and the binding sites of regulators, which may result in the different production levels of chloramphenicol and jadomycin. These genomic differences can be used as rational engineering targets for increasing the productivity of a given secondary metabolite in *Streptomyces*. Besides, our data shows that not only are the production levels of chloramphenicol and jadomycin different among the strains but also that chloramphenicol and jadomycin productions are regulated in an antagonistic manner. Elucidating the different secondary metabolite production in closely related strains would enable us to better understand the complicated secondary metabolism and improve the production levels for possible large-scale usage.

## 2. Materials and Methods

### 2.1. Bacterial Strains and Cell Growth

Ten *S. venezuelae* strains were purchased from the American Type Culture Collection (ATCC 10712, ATCC 10595, ATCC 21113, ATCC 15439, ATCC 15068, ATCC 14583, ATCC 14584, ATCC 14585, ATCC 21018, and ATCC 21782). The cells were initially propagated on ISP2 agar medium (4 g/L yeast extract, 10 g/L malt extract, 4 g/L glucose, and 20 g/L agar). Then, a single colony of each strain was inoculated in 50 mL of ISP2 medium with 8 g of glass beads (3 ± 0.3 mm diameter) in a 250 mL baffled flask and cultivated at 30 °C in a shaking incubator at 200 rpm. Cell stocks were prepared with 50% glycerol and then stored at −80 °C. Cells were cultured in MYM medium (4 g/L yeast extract, 10 g/L malt extract, and 4 g/L maltose) for genomic DNA (gDNA) extraction and production of chloramphenicol and jadomycin.

### 2.2. Screening S. venezuelae Strains Harboring Chloramphenicol BGCs

The chloramphenicol BGCs in *S. venezuelae* strains were identified using the gene-specific PCR. PCR was carried out using TaKaRa LA Taq^TM^ polymerase with GC buffer (TaKaRa, Shiga, Japan) in a final volume of 20 µL. The reaction mixture consisted of 120 ng of gDNA, 1 unit of TaKaRa LA Taq^TM^ polymerase, 10 µL of 2× GC buffer II, 3.2 µL of 2.5 mM dNTP mixture, and 5 pmol of each primer. The primer sequences are available inTable S2. PCR reactions were performed on a C1000 thermal cycler (Bio-Rad, Hercules, CA, USA) programmed as follows: an initial denaturation step at 94 °C for 1 min, followed by 30 cycles at 94 °C for 30 s, 60 °C (*cmlB*, *cmlP*) or 62 °C (*cmlH*) for 30 s, 72 °C for 1 min 30 s, with a final extension at 72 °C for 5 min.

### 2.3. Genome Sequencing of Three S. venezuelae Strains

For gDNA extraction, the cultured cells were harvested at the mid-exponential phase and washed twice with 10 mM EDTA. To weaken the cell wall, the harvested cells were treated with 10 mg/mL lysozyme at 37 °C for 45 min. Then, the gDNA was extracted using a Wizard Genomic DNA Purification Kit (Promega, Madison, WI, USA) according to the manufacturer’s instruction. PacBio genome sequencing library was prepared using 5 µg of gDNA by using SMRTbell^TM^ and then sequenced using the PacBio RS II sequencing platform (Pacific Biosciences, Menlo Park, CA, USA) [24]. For constructing the Illumina HiSeq sequencing library, the gDNA was fragmented to approximately 350 bp using the S220 Focused-ultrasonicator (Covaris Inc., Woburn, MA, USA) and used to construct the sequencing library using TruSeq DNA PCR-Free LT kit (Illumina Inc., San Diego, CA, USA). The prepared library was sequenced on the HiSeq 2500 with a 100 bp single-read running scale.

The obtained PacBio sequencing reads were de novo assembled using the hierarchical genome assembly process (HGAP, version 2.3 (Pacific Biosciences, Menlo Park, CA, USA)) [25]. Illumina sequencing reads were first quality trimmed by CLC genomic workbench (CLC Bio, Aarhus, Denmark) with default parameters (ambiguous trim limit: 2; quality trim limit: 0.05) and assembled using the de novo assembly function of CLC genomic workbench. Then, the high-quality complete genomes were constructed by hybrid genome assembly and genome correction, as described previously [26]. The three complete genomes were annotated using the National Center for Biotechnology Information (NCBI)’s Prokaryotic Genome Annotation Pipeline. Genome sequences are available at NCBI under the accession numbers CP029197 (ATCC 10712), CP029196 (ATCC 21113), and CP029195 (ATCC 10595).

### 2.4. Comparative Genome Analysis

To investigate the difference between the previously reported genome (FR845719) and the newly completed genome (CP029197), the sequence of FR845719 was aligned to the CP029197 using the CLC genomic workbench. Before alignment, the sequence of FR845719 was fragmented, starting with the undetermined sequence “N” to identify the position of the unassembled sequence due to the “N”. The mapping parameters are as follows. Mismatch cost: 2; Insertion cost: 3; Deletion cost: 3; Length fraction: 0.2; Similarity fraction: 0.5; Non-specific match handling: Map randomly. The length of misassembled sequences at the left or right side of each fragment and the length of overlaps or gaps between adjacent fragments were calculated using the SAM formatted mapping file. Next, the coding sequences of FR845719 were mapped to CP029197 using the same parameter to check the annotation changes between the two genome sequences. We then categorized annotated genes of CP029197 according to the 5′ and 3′ end of the genes of FR845719 using Perl scripts. If two genes from each genome had identical 5′ and 3′ ends, the gene of CP029197 was categorized as the “Identical” category. If the gene of CP029197 was longer than the corresponding gene of FR845719, it was in the “Long” category, and the gene was shorter than the annotation of FR845719, it was categorized in the “Short” class. When both the 5′ and 3′ end of two genes did not match and two genes overlapped over 50% of the length, the gene of CP029197 was divided into the “Moved” category. The newly annotated gene in CP029197 was categorized as “New” class. A “Missing” gene indicated that it was found only in FR845719.

### 2.5. Genome Analyses

For phylogenetic analysis, the average nucleotide identity based on MUMmer algorithm (ANIm) values was calculated between the complete genomes using Python module PYANI (https://github.com/widdowquinn/pyani) [27]. The complete genomes of 121 *Streptomyces* species, including NRRL B-65442 (NZ_CP018074.1) and ATCC 15439 (CP013129.1), and *Mycobacterium tuberculosis* H37Rv (NC_000962.3) were downloaded from NCBI. The orthologous clusters were predicted by OrthoVenn2 [28] using the protein sequence files of which pseudogenes were excluded. The E-value cut off of 1 e^−5^ and an inflation value of 1.5 were used to calculate sequence similarities and define orthologous genes, respectively. The predicted smBGCs were obtained by antiSMASH [29], and the smBGCs and corresponding genomic regions were visually compared using EasyFig [30]. The smBGCs from the strains were compared, and their conserveness was analyzed. If the predicted core biosynthetic genes from the two smBGCs are aligned, these smBGCs were categorized as “Conserved”. If not, the smBGC was categorized as “Missing”. In the case of smBGCs from *S. venezuelae* ATCC 15439, more criteria were required to be categorized. When a single type of smBGC from ATCC 15439 is aligned to a single region that contains two types of smBGCs from ATCC 10712, the smBGC was categorized as “Partially conserved”. When the accessory genes of smBGC other than the core biosynthetic genes were conserved, the smBGC was also categorized as “Partially conserved”. The smBGCs that only predicted in the genome of ATCC 15439 were categorized as “15439-specific”. When one of two types of smBGCs from ATCC 15439 was conserved in a single type of smBGC from ATCC 10712, the non-aligned cluster was classified as “15439-specific”. When conserved smBGCs were predicted at the 5′ or 3′ end of the genome, respectively, the smBGCs were categorized as “Conserved in other regions”.

### 2.6. Measurement of Chloramphenicol and Jadomycin

*S. venezuelae* strains were pre-cultured in MYM liquid medium, and these pre-cultured cells were inoculated in the new media with an initial optical density of 0.01 (600 nm). After incubation for 36 h, 1 mL of culture broth was extracted with two volumes of ethyl acetate. The organic phase was collected and evaporated, which was then dissolved with 500 µL of methanol. A volume of 10 µL of the extract was analyzed by a Triple Quad 3500 (SCIEX, Framingham, MA, USA) equipped with an ESI source and a Nexera X2 UHPLC system (Shimadzu, Kyoto, Japan). UHPLC analyses were performed on an Agilent ZORBAX Eclipse Plus C18 column using a gradient condition of 80:20 (acetonitrile with 0.1% formic acid: water with 0.1% formic acid) over 4 min at a flow rate of 0.5 mL/min followed by an increase to 95:5 over 0.5 min. The condition was held for 0.5 min, followed by a decreasing linear gradient to 80:20 over 0.1 min, and an ending with the gradient of 80:20 over 0.9 min. Chloramphenicol was determined by negative ion mode and quantified by a selected product ion, 151.5 Da. Data processing was performed in Analyst^®^ 1.6.3 (SCIEX, Framingham, MA, USA). A volume of 100 µL of the remaining elution sample was then measured by absorbance at 526 nm with a BioTek Synergy H1 (BioTek Instruments, Inc., Winooski, VT, USA) to estimate the jadomycin production [31,32]. All measurements were performed with biological duplicates and technical duplicates. In the case of ethanol shock experiment, 100% ethanol or autoclaved distilled water was added 6 h after inoculation, and cells were harvested 30 h after ethanol shock (a total of 36 h of incubation).

### 2.7. Engineering of S. venezuelae ATCC21113 Using CRISPR/Cas9 System

For the insertion of a cytosine nucleotide at position 505 of *cmlI* of ATCC 21113, a 2-kb editing template was amplified from the gDNA of ATCC 21113 using Phusion polymerase with Editing_F and Editing_R primers (Table S3). The PCR amplicon was cloned into the pJET1.2/blunt cloning vector (Thermo Fisher Scientific Inc., Waltham, MA, USA). Next, site-directed mutagenesis was performed using this vector as the template using the SDM_F and SDM_R primers (Table S3). The insertion was confirmed by Sanger sequencing using Sanger_check oligo (Table S3), and the C-inserted editing template was cloned into the XbaI site of the pCRISPomyces-2 vector, which contains a sgRNA cassette [33]. The constructed vector was transformed into ET12567/pUZ8002 for demethylation, and the final demethylated vector was transformed to ATCC 21113 using the protoplast method [34]. The obtained transformants were streaked onto MYM agar plate containing 50 µg/mL of apramycin and incubated at 30 °C. The transformants were cultured again on the MYM agar plate without apramycin and incubated at 37 °C twice to cure the vector, followed by sporulation to obtain the single transformant. Finally, the gDNA of the constructed apramycin sensitive strain was extracted and amplified using NF_check_F and NF_check_R primers (Table S3), and confirmed by Sanger sequencing.

## 3. Results

### 3.1. Screening S. venezuelae Strains Harboring Chloramphenicol BGC

**Figure 1:**
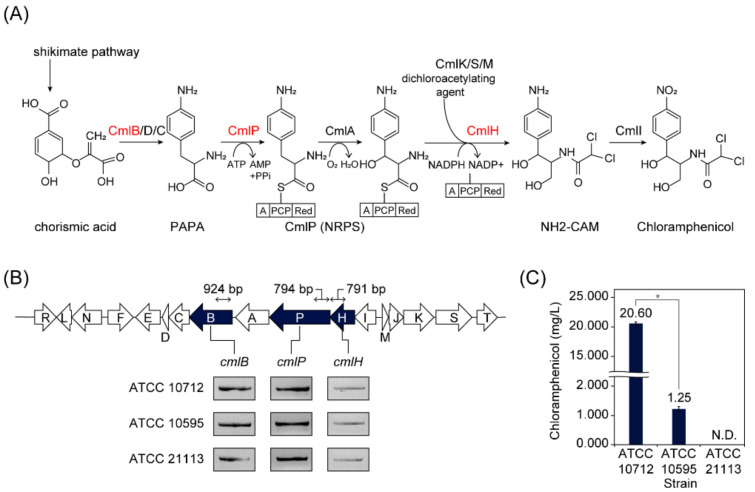
Screening *S. venezuelae* strains harboring chloramphenicol biosynthetic gene cluster (BGC). (**A**) Biosynthetic pathway of chloramphenicol. The three selected genes for PCR are colored as red. PAPA, L-*p*-aminophenylalanine; NRPS, non-ribosomal peptide synthetase; NH2-CAM, D-*threo*-1-(4-aminophenyl)-2-dichloroacetylamino-1,3-propanediol. (**B**) Organization of chloramphenicol BGC and detection of the three genes from the three *S. venezuelae* strains (ATCC 10712, ATCC 10595, and ATCC 21113). The three selected genes for PCR are colored navy. (**C**) Chloramphenicol production of the three *S. venezuelae* strains. N.D., Not detected. An asterisk indicates *p*-value < 0.05 (Wilcoxon rank-sum test).

*Streptomyces* possess diverse smBGCs in their genomes and showed different metabolite profiles on the strain level. For example, chloramphenicol was initially isolated from the culture of strain ATCC 10712 [6], and later, ATCC 10595 and NRRL B-65442 were reported to produce chloramphenicol as well [17,35]. However, comparisons of chloramphenicol production between *S. venezuelae* strains had not been done, which could suggest rational reverse engineering strategies for enhancing secondary metabolite production. To compare the chloramphenicol production on the strain level, we sought to identify additional *S. venezuelae* strains harboring chloramphenicol BGC. To this end, we screened the *S. venezuelae* strains by employing the gene-specific PCR using primers specific to *cmlB*, *cmlP*, and *cmlH* genes, which are the main biosynthetic genes of the chloramphenicol BGC (Figure 1A; Table S2). Briefly, *cmlB* gene encodes 4-amino-4-deoxychorismate synthase, which converts chorismic acid to 4-amino-4-deoxy chorismic acid [36]. A non-ribosomal peptide synthetase (NRPS) encoded by *cmlP* gene recognizes and loads L-*p*-aminophenylalanine (PAPA) onto the phosphopantetheine arm of the peptidyl carrier protein domain [37]. A protein encoded by the *cmlH* gene is predicted to transfer the dichloroacetyl group to the intermediate of chloramphenicol [38,39]. Among the ten *S. venezuelae* strains available at the American Type Culture Collection, ATCC 10712 and ATCC 10595 had all three genes, as expected. Additionally, ATCC 21113, which was known to produce glucose isomerase enzyme, was newly found to have these three chloramphenicol biosynthetic genes in its genome (Figure 1B). Next, we measured the chloramphenicol production levels of the three strains (ATCC 10712, ATCC 10595, and ATCC 21113). After 36 h of growth in liquid MYM medium, the chloramphenicol of ATCC 10712 (20.60 mg/L ± 0.27) was 16.5 times higher than that of ATCC 10595 (1.25 mg/L ± 0.07), whereas no production of chloramphenicol was observed for ATCC 21113 (Figure 1C). These results led us to sequence their genomes to investigate the genomic differences, which may affect the different chloramphenicol productions.

### 3.2. Genome Completion of the Three S. venezuelae Strains Harboring Chloramphenicol BGC

To reveal the differences in chloramphenicol production observed between *S. venezuelae* strains, we sought to compare the genomes of the three strains. Among the strains, the genome sequences of ATCC 10595 and ATCC 21113 have never been reported. In the case of ATCC 10712, a genome sequence was reported (Accession number: FR845719), but it contained a total of 9156 undetermined sequences, including 9150 ‘N (any nucleotide)’s, 2 ‘M (A or C)’s, 3 ‘S (C or G)’s, and ‘K (G or T)’ (Figure S1A). Considering the importance of the strain as an emerging model species of *Streptomyces*, we decided to complete the genome of ATCC 10712 newly. Therefore, the complete genomes of the three strains were de novo assembled by a hybrid assembly strategy of long-read sequencing (PacBio RS II) and short-read sequencing (Illumina) [26]. The lengths of the final complete genomes were 8.22 Mbp (ATCC 10712), 7.87 Mbp (ATCC 10595), and 7.89 Mbp (ATCC 21113), respectively, and all genomes had a 72.5% G + C content (Table 1). These are comparable to the previously reported genomes of *S. venezuelae* ATCC 15439 (9.05 Mbp, 71.7% G + C content) and *S. venezuelae* NRRL B-65442 (8.38 Mbp, 72.4% G + C content). In the newly completed genomes, the number of annotated CDSs was 7377 (ATCC 10712), 6942 (ATCC 10595), and 6987 (ATCC 21113). Twenty-one rRNAs and 67 tRNAs were annotated in each of the three complete genomes. Compared to the previously reported genome sequence of ATCC 10712 (FR845719) containing numerous ambiguous sequences, in the newly completed genome sequence of the strain (CP029197), a total of 147 gaps (a total of 33,025 bp) were filled and 46 overlapping regions (a total of 6947 bp) were corrected (Figure S1B, Table S4).

**Figure 2:**
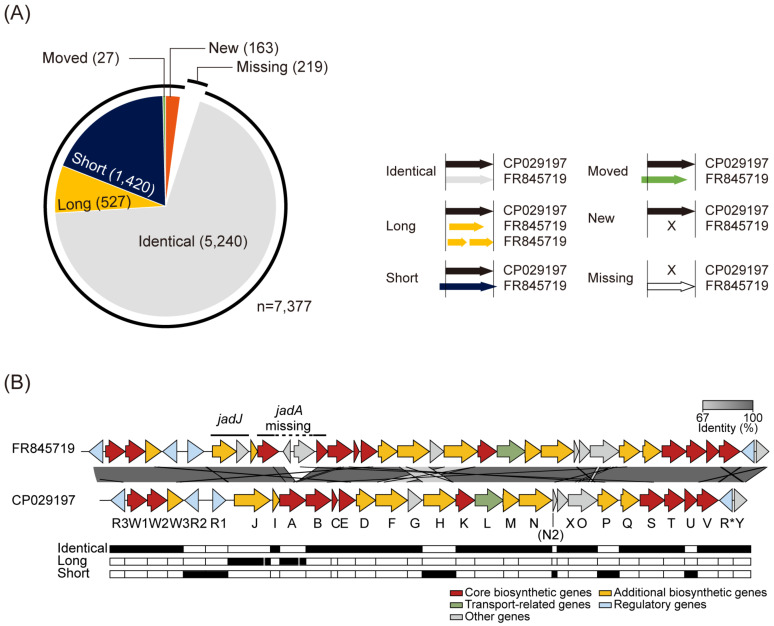
High-quality genome completion of *S. venezuelae* ATCC 10712. (**A**) Corrected gene annotation of CP029197 compared to that of FR845719. The annotated genes of CP029197 were categorized according to the 5′ and 3′ ends of the genes of FR845719. (**B**) Alignment of jadomycin BGC from the previously reported genome of ATCC 10712 (FR845719) and the newly completed genome (CP029197). The genes were colored according to the antiSMASH prediction. Gray bars between the clusters show the BLASTn identity value.

High-quality genome sequences could be utilized to identify the genes in smBGCs accurately. The annotated genes in CP029197 were categorized according to the relative positions of the corresponding genes annotated in FR845719 (Figure 2A). In total, 5240 genes were identical in both genomes, whereas 1420, 527, and 27 genes were assigned to ‘Short’, ‘Long’, and ‘Moved’ categories, respectively. Interestingly, 163 genes were newly annotated in CP029197, and 219 genes of FR845719 were not annotated in CP029197. For example, the smBGC for jadomycin biosynthesis contains 35 genes in FR845719, of which 23 genes were identical, six genes were shortened, four genes were lengthened, and two genes were absent in CP029197 (Figure 2B). In the case of *jadJ*, categorized as ‘Long’, two separate genes (SVEN_5974 and SVEN_5975) were annotated in FR845719 (Figure S1C). However, it has been previously identified that *jadJ* exists as a single gene by dideoxynucleotide sequencing, which sequence is available at NCBI under the accession number AF126429 [40,41]. This difference comes from the annotation of the upstream gene without a stop codon. Two genes (SVEN_5977 and SVEN_5980) from the initial assembly were both identified as part of a single gene in the new assembly, called *jadA*. Between the two genes, a 2201 bp region containing two additional genes (SVEN_5978 and SVEN_5979) exists in FR845719, which seems to be misassembled because of 20 ‘N’s in SVEN_5977 gene (Figure S1C). In addition, between the *jadN* and *jadX* genes, an additional coding sequence, whose function was predicted as an acyl-CoA carboxylase subunit epsilon, was newly annotated as *jadN2* (Figure 2B). Its function seemed to be closely related to the *jadN,* which is an acyl-CoA carboxylase subunit beta. Taken together, this accurate genome annotation for ATCC 10712 based on the high-quality complete genome sequence precisely determines the genes in smBGCs that could be used as great resources for further studies of secondary metabolite biosynthesis.

### 3.3. Genomic Divergence between S. venezuelae Strains

**Figure 3:**
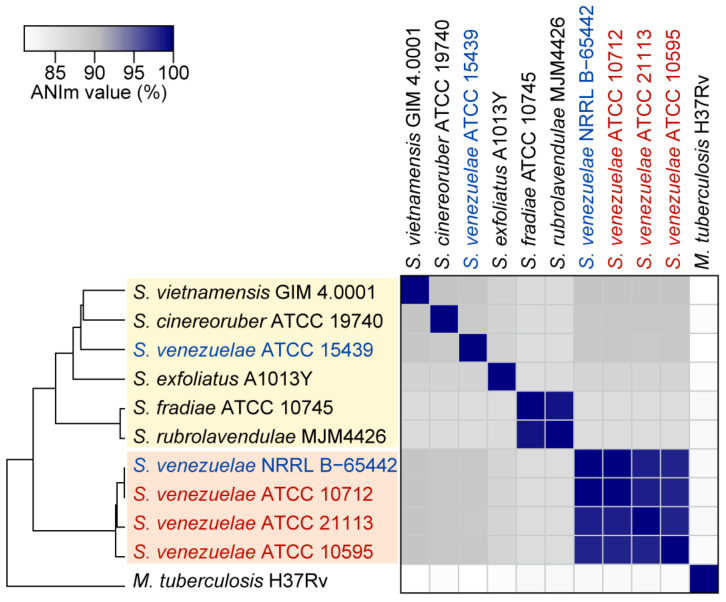
The average nucleotide identity values based on MUMmer algorithm (ANIm) between *S. venezuelae* strains and other *Streptomyces* species. The four closely related *S. venezuelae* strains (ATCC 10712, NRRL B-5442, ATCC 21113, and ATCC 10595) are in the orange box. The strains that were clustered with *S. venezuelae* ATCC 15439 are in the yellow box. In particular, *S. venezuelae* strains whose genome sequences were completed in this study are represented as red, and the other *S. venezuelae* strains are represented as blue. *Mycobacterium tuberculosis* H37Rv was used to show the phylogenetic distance with other species.

To infer the genomic distance between *S. venezuelae* strains, we calculated the average nucleotide identity (ANI) value of the three complete genomes, and the two genomes reported at the NCBI database (NRRL B-65442 and ATCC 15439) (Figure 3). In particular, average nucleotide identity based on MUMmer algorithm (ANIm) values were calculated because they can provide more robust results than the ANI-based BLAST algorithm when the comparing genomes are highly similar [42]. NRRL B-65442, ATCC 21113, and ATCC 10595 had a 99.9%, 98.5%, and 98.2% ANIm value with ATCC 10712, respectively. Meanwhile, ATCC 15439 had a low ANIm value with the other four strains (89.0% on average). Indeed, a 95% ANI value is regarded as the general threshold for classifying organisms as the same species [42]. Therefore, considering that the ATCC 10712 is the type strain of *S. venezuelae*, ATCC 21113, ATCC 10595, and NRRL B-65442 could be classified as the *S. venezuelae* species, and ATCC 15439 was a distinct species from *S. venezuelae*. Although ATCC 10712 and NRRL B-65442 were the most closely related strains, NRRL B-65442 had an additional 158-kb linear plasmid.

The ANIm analysis with the previously reported complete genomes of other 116 *Streptomyces* species showed that the four *S. venezuelae* strains having chloramphenicol BGC were highly related, and the four strains were phylogenetically distant from other *Streptomyces* species (Figure 3, Figure S2). In the case of ATCC 15439, it had the highest ANIm value with *S. vietnamensis* GIM4.001 (89.3%), followed by four *S. venezuelae* strains (89.0% on average) and *S. cinereoruber* ATCC 19740 (88.9%). Thus, ATCC 15439 could be designated as a new species. However, the strain has similar characteristics to ATCC 10712, such as a fast growth rate, sporulation in the liquid medium, and ease of genetic manipulation [19].

**Figure 4:**
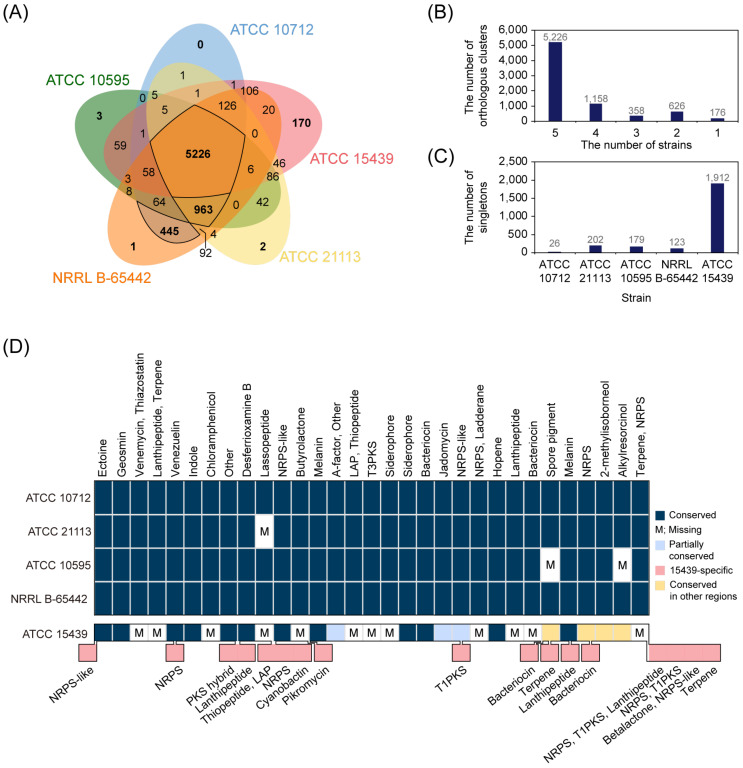
Genome comparison of *S. venezuelae* strains. (**A**) The distribution of orthologous genes among the five *S. venezuelae* strains. (**B**) The number of orthologous genes according to the number of strains. (**C**) The number of singletons of each strain. (**D**) The predicted secondary metabolite biosynthetic gene clusters (smBGCs) of *S. venezuelae* strains. NRPS, non-ribosomal peptide synthetase; LAP, linear azol(in)e-containing peptides; T3PKS, type III polyketide synthase; PKS, polyketide synthase; T1PKS, type I polyketide synthase.

To investigate the differences in coding regions among the five *S. venezuelae* strains (ATCC 10712, ATCC 21113, ATCC 10595, NRRL B-65442, and ATCC 15439), protein sequences from the strains were compared using OthoVenn2 [28], which compared all input protein sequences and predicted orthologous genes that were conserved among the selected organisms. There were 5226 orthologous genes shared by the five strains (Figure 4A,B). In addition, four closely related *S. venezuelae* strains shared 963 additional orthologous genes, resulting in a total of 6189 core orthologous genes among the four strains. These genes accounted for 86.9% (ATCC 10712), 90.9% (ATCC 21113), 92.2% (ATCC 10595), and 85.4% (NRRL B-65442) of the total protein-coding genes of each strain, indicating that most of the protein-coding genes in the four strains were conserved. In particular, ATCC 10712 and NRRL B-65442 shared 445 additional orthologous genes, whose location was particularly concentrated in the range of 6 to 6.3 Mbp and the region was not presented in the genomes of ATCC 21113 and ATCC 10595 (Figure S3A,B). In addition, 26.3% of the orthologous genes between ATCC 10712 and NRRL B-65442 were located at 2.1–2.4 Mbp, indicating that the variations were grouped in several regions in the genome, not limited to the end of the genome. Compared to the other strains, ATCC 15439 had many strain-specific genes, including 170 inparalogs that present as orthologous genes in a single strain and 1912 singletons that present as a single gene in a single strain (Figure 4C). In sum, ATCC 15439 showed a difference in gene contents from other *S. venezuelae* strains, whereas closely related *S. venezuelae* strains shared more than 85.4% of total genes.

### 3.4. Highly Conserved smBGCs among S. venezuelae Strains

To explore the conservation and divergence of smBGCs in the *S. venezuelae* strains, we predicted smBGCs using antiSMASH (Table S5). The number of predicted smBGCs was 30 in the three genomes (ATCC 10712, ATCC 21113, and NRRL B-65442), whereas ATCC 10595 and ATCC 15439 genomes contained 27 and 31 smBGCs, respectively. The predicted types of smBGCs and their distribution along the chromosomes were almost identical in the four closely related strains, except for ATCC 15439 (Figure 4D). Based on the antiSMASH prediction, a lasso peptide BGC was absent in ATCC 21113 (Figure S4A), whereas a lanthipeptide BGC was only found in ATCC 21113 compared to other closely related strains. However, we found that the three other strains have the same lanthipeptide BGC in their genomes by using the Protein BLAST (Figure S4B). Therefore, we concluded that the lanthipeptide BGC was not solely present in the genome of ATCC 21113, but also in the rest of the three strains (Table S6). In the case of ATCC 10595, two smBGCs were absent compared to the other three strains. The two lost smBGCs were a spore pigment BGC (Type II polyketide synthase (PKS)) and an alkylresorcinol BGC (Type III PKS) (Figure S4C). Except for these differences, all predicted smBGCs were identical in all the four *S. venezuelae* strains. Notably, the BGCs of chloramphenicol and jadomycin (predicted as rabelomycin), which are widely studied valuable secondary metabolites in ATCC 10712, were found in all four strains.

Next, pairwise analysis of the predicted smBGCs between ATCC 10712 and ATCC 15439 revealed that 16 smBGCs were conserved and three smBGCs were partially conserved between the two strains. The conserved smBGCs include ectoine, geosmin, 2-methylisoborneol, hopene, siderophore, and melanin, which are commonly found in the *Streptomyces* species. Otherwise, 12 smBGCs were ATCC 10712-specific and the other smBGCs were ATCC 15439-specific (Figure 4D, Table S6). Of the 16 conserved smBGCs, the genomic locations of 12 smBGCs were similar, whereas the other four smBGCs were located in different regions near the 3′ or 5′ end of the two genomes (spore pigment, NRPS, 2-methylisoborneol, and alkylresorcinol). It showed that conserved smBGCs were also relocated within the genome after separation of the lineages. In the case of the three partially conserved smBGCs, several genes or surrounding genes of the matched known cluster were aligned. For example, five regulatory genes (*jadR3*, *jadW1*, *jadW2*, *jadW3*, and *jadR2*) of jadomycin BGC were present in the butyrolactone BGC of ATCC 15439, but other jadomycin biosynthetic genes were absent. Considering that regulators in an smBGC often affect the functional expression of other smBGCs [43], these regulatory genes are likely to regulate the expression of other smBGCs of ATCC 15439. These partially conserved smBGCs represented traces of past cluster presence or could be acquired via horizontal gene transfer [44,45]. Taken together, most of the smBGCs were highly conserved in the four closely related strains, except for ATCC 15439 that was characterized by having many strain-specific smBGCs, including pikromycin BGCs.

### 3.5. Comparison of Chloramphenicol BGCs of S. venezuelae Strains

**Figure 5:**
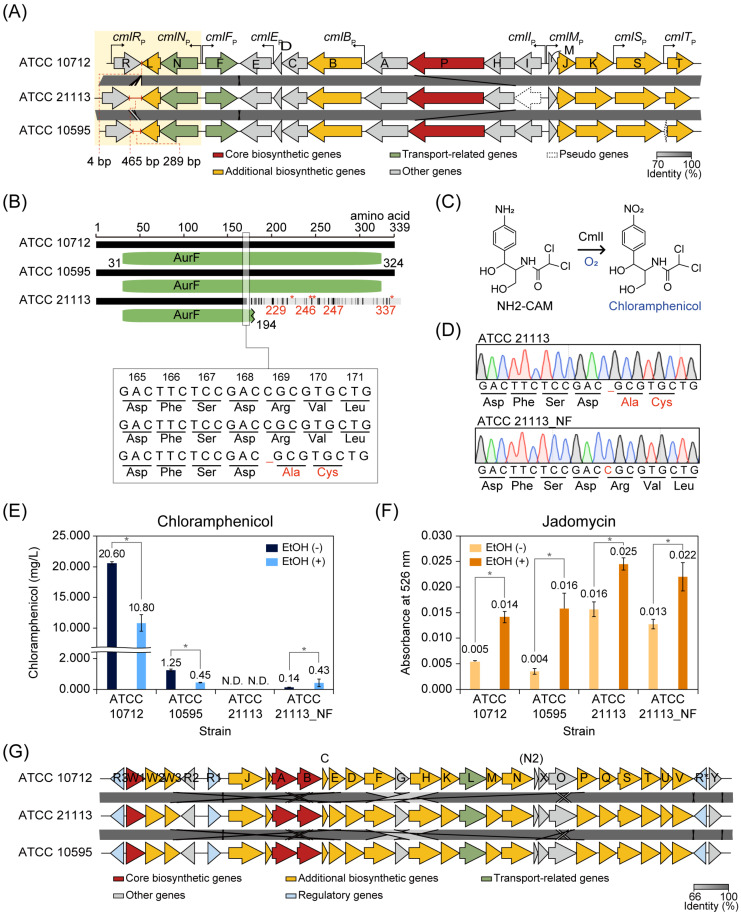
Comparison of chloramphenicol and jadomycin production. (**A**) Alignment of the chloramphenicol biosynthetic gene cluster (BGC) from the three *S. venezuelae* strains. The region of *cmlR* and *cmlLN* operon was represented with a yellow box, and the red line indicates the intergenic region of *cmlR* and *cmlL*. The genes were colored according to the classification of antiSMASH prediction. Gray bars between the clusters indicate BLASTn identity value. The chloramphenicol biosynthetic genes were named, according to Sekurova et al. [50]. (**B**) Comparison of amino acid sequences of CmlI from the three *S. venezuelae* strains. Amino acid 31 to 324 of the CmlI was predicted as the AurF domain by Pfam [51], and it was represented as a green rounded rectangle. The asterisk indicates the position of a stop codon. (**C**) N-oxygenation of NH2-CAM by CmlI to produce chloramphenicol. (**D**) Sanger sequencing results of the *cmlI* gene from ATCC 21113 and ATCC 21113_NF. (**E**) Chloramphenicol production of the three *S. venezuelae* strains and ATCC 21113_NF in the absence or presence of ethanol. EtOH, ethanol; N.D., Not detected. Asterisk indicates *p*-value < 0.05. (Wilcoxon rank-sum test) (**F**) Jadomycin production of the three *S. venezuelae* strains and ATCC 21113_NF in the absence or presence of ethanol. Absorbance at 526 nm was measured for estimating jadomycin production. EtOH, ethanol; Asterisk indicates *p*-value < 0.05. (Wilcoxon rank-sum test) (**G**) Alignment of the jadomycin BGC from the three *S. venezuelae* strains. The genes were colored according to the prediction of antiSMASH. Gray bars between the clusters indicate BLASTn identity value. The jadomycin biosynthetic genes were named, according to Niu et al. [52].

*S. venezuelae* ATCC 10712, ATCC 21113, and ATCC 10595 had chloramphenicol BGC, and the overall genomic similarity between the three strains was high. However, the three strains showed a large difference in chloramphenicol production (Figure 1C). To understand this, we compared the chloramphenicol BGCs from these strains. The overall alignment of genes in each chloramphenicol BGC of the three strains revealed that a cytosine nucleotide was deleted at position 505 in the *cmlI* of ATCC 21113, compared to the other strains (Figure 5A,B). The deletion mutation led to a frameshift and amino acid changes from amino acid 169 in the CmlI (Figure 5B). The CmlI introduces an oxygen atom into the aryl-amine analog of chloramphenicol, D-*threo*-1-(4-aminophenyl)-2-dichloroacetylamino-1,3-propanediol (NH2-CAM), to yield the aryl-nitro group of the active antibiotic [46], which is the final step in chloramphenicol biosynthesis [47] (Figure 5C). Therefore, we suggest that ATCC 21113 could not produce chloramphenicol due to the functional loss of the *cmlI* gene. To confirm this, ATCC 21113 was engineered by inserting a single cytosine at position 505 of the *cmlI* gene using CRISPR/Cas9 system to correct the frameshift mutation and produce chloramphenicol (Figure 5D). The engineered strain, ATCC 21113_NF, produced a small amount of chloramphenicol (0.14 mg/L ± 0.01) compared to ATCC 10712 (20.60 mg/L ± 0.27) and ATCC 10595 (1.25 mg/L ± 0.07) (Figure 5E). The chloramphenicol production of ATCC 21113_NF was still more repressed than in other strains.

As ATCC 21113 and ATCC 10595 produced lesser amounts of chloramphenicol than ATCC 10712, the sequence variations in chloramphenicol BGCs between the three strains could give insights into the different production levels. Only three protein sequences (CmlE, CmlD, and CmlM) were completely conserved in the three strains. In the remaining thirteen protein-coding genes, a total of 126 sites of amino acids were different between the strains (Figure S5A, Table S7). Similar to *cmlI* gene, five genes (*cmlR*, *cmlN*, *cmlC*, *cmlA*, and *cmlK*) include indel mutations between the strains, but there were no consequent frameshift mutations. Among these, one of the differences observed in both ATCC 21113 and ATCC 10595 was the additional six or four amino acid insertions in the linker region of the N-terminal domain and the metallo-β-lactamase domain of CmlA. This extended linker region may affect the structural change and enzymatic activity of CmlA.

Moreover, intergenic regions were also different between the strains. The length of the intergenic sequence between *cmlR* and *cmlL* was 4 bp, 465 bp, and 289 bp in ATCC 10712, ATCC 21113, and ATCC 10595, respectively (Figure 5A). In ATCC 10712, this region was suggested to have a regulatory role as a cutoRNA (convergent untranslated overlapping RNAs) because the 3′ untranslated region of the *cmlR* and *cmlLN* operon transcript extends throughout the coding regions of each other [48]. Considering that *cmlR* is a transcriptional activator of the chloramphenicol BGC and *cmlN* is predicted to export chloramphenicol [39], it was expected that the cutoRNA has a regulatory role of production and export of chloramphenicol. In addition, the alignment of the seven upstream regions and intergenic regions detected the difference in specific tandem repeats in the three upstream regions (Figure S5B). In the upstream region of the *cmlR* gene, the ‘CCCGTTTC’ sequence was repeated four times and two times in ATCC 21113 and ATCC 10712, respectively, while there was no repeat in ATCC 10595. The ‘GCCGGAGGGGCCG’ sequence in the intergenic region of *cmlB* and *cmlA* was repeated two times in ATCC 10712 and ATCC 10595, whereas there were no repeats of this sequence in ATCC 21113. In the intergenic region of *cmlI* and *cmlM*, the ‘TTCCCTTTGCGGGCTC’ sequence was repeated two times in ATCC 10712 and ATCC 21113, but not for ATCC 10595. These tandem repeats can modify the binding affinity of regulatory proteins [49]. Therefore, these differences between the three strains may affect the regulation of chloramphenicol biosynthesis. In all, ATCC 10712, ATCC 10595, and ATCC 21113 showed different chloramphenicol production. In particular, ATCC 21113 could not produce chloramphenicol due to the indel mutation in the *cmlI* gene. Interestingly, ATCC 21113_NF, which corrected its frameshift mutation in the *cmlI* gene, produced a small amount of chloramphenicol, compared to the other two strains.

### 3.6. Comparison of Jadomycin BGCs of S. venezuelae Strains

The jadomycin BGC was also conserved in the four closely related *S. venezuelae* strains. However, jadomycin production has been conducted only in ATCC 10712 [8,31,53,54]. Under normal MYM culture conditions, the jadomycin biosynthesis levels of ATCC 21113 and ATCC 21113_NF were higher than those of ATCC 10712 and ATCC 10595 (Figure 5F). As ethanol shock (ES) treatment is a common practice to induce the jadomycin production in ATCC 10712 [54], we measured the jadomycin production levels of the closely related strains in response to ES treatment. As expected, jadomycin production was significantly increased in the four strains under the ES condition (Wilcoxon rank-sum test, *p*-value < 0.05) (Figure 5F). Among the strains, ATCC 21113 and ATCC 21113_NF still showed higher jadomycin production levels than ATCC 10712 and ATCC 10595. In contrast, chloramphenicol production levels were reduced by 1.9- and 2.8-fold in ATCC 10712 and ATCC 10595, respectively, compared to the normal MYM culture condition (Figure 5E). This result indicates that the jadomycin and chloramphenicol biosynthesis were regulated antagonistically upon ES treatment, which is consistent with the results in the galactose–isoleucine medium that ES treatment induces jadomycin biosynthesis and inhibits chloramphenicol biosynthesis [43]. However, in ATCC 21113_NF, ES treatment stimulated the chloramphenicol production (3.1-fold, *p*-value < 0.05), whereas less change was observed in jadomycin production.

Comparing the protein sequences of 31 genes in the jadomycin BGC, protein sequences of nine genes (*jadR2*, *jadR1*, *jadI*, *jadA*, *jadC*, *jadE*, *jadD*, *jadT*, and jadR*) were identical in ATCC 21113, ATCC10712, and ATCC 10595 strains (Figure S6A, Table S8). In particular, the sequences of regulatory proteins known to control the jadomycin or chloramphenicol biosynthesis were identical, excluding JadR3, which showed a single amino acid difference at the 3′ end. Therefore, we speculated that variations of the sites where the regulatory proteins bind would affect the different expression of genes in the jadomycin BGC and result in different levels of jadomycin production. The regulatory sites to which JadR3 binds, including AREI, AREII, and AREIII in the intergenic region of *jadR2* and *jadR1*, and AREIV in the intergenic region of *jadR3* and *jadW1*, were conserved among all three strains (Figure S7B). However, there were many variations in the regions between *jadR2* and *jadR1*, where many regulators are known to bind. Of the regulatory protein binding sites, variations were found at the regions known as the binding sites of JadR2 and JadR*. Both JadR2 and JadR* repress the transcription of *jadR1*, the activator of jadomycin BGC [55]. In particular, single nucleotide and deletion mutations were found at the JadR2 binding site in ATCC 21113, where specific inverted repeats exist. In the case of JadR3, it has been reported that if a mutation happens in an inverted repeat of the AREII binding site, it cannot bind to the AREII binding site [56]. Therefore, the mutation in this inverted repeat can weaken the binding of JadR2, and thus the repression of the transcription of *jadR1* in the ATCC 21113 may be weakened. Taken together, the closely related *S. venezuelae* strains showed differences in jadomycin and chloramphenicol production. ATCC 21113 produced the highest amount of jadomycin among the three *S. venezuelae* strains. The ATCC 21113_NF, which was engineered to produce chloramphenicol, was able to produce jadomycin like the wild-type ATCC 21113. We also showed that the biosynthesis of chloramphenicol and jadomycin are regulated in an antagonistic manner upon ES treatment.

## 4. Discussion

In this study, we provided high-quality genomes of three *S. venezuelae* strains, including type strain ATCC 10712, ATCC 21113, and ATCC 10595. The comparative genomic analyses with reported genomes of other *S. venezuelae* strains (NRRL B-65442 and ATCC 15439) revealed that the four strains, except the ATCC 15439 strain, showed high genomic similarities (>98% ANIm), indicating that they could be regarded as the same species. In these four strains, 85.4%–92.2% of the total genes belonged to the orthologous genes, and most of the smBGCs were conserved. This result is similar to the case of *S. pratensis* strains, of which 85%–88% of the genes were shared by the strains, and all smBGCs were conserved [57]. Meanwhile, ATCC 15439 could be classified as a distinct species from other *S. venezuelae* species according to the ANIm analysis, and its genome encodes many strain-specific smBGCs. Several reclassifications of *Streptomyces* have been reported for species-level using a whole-genome comparison approach [58,59].

Despite most of the smBGCs being conserved among the closely related *S. venezuelae* strains, the production of specific secondary metabolites—chloramphenicol and jadomycin—differed among the strains. The different secondary metabolite profiles of phylogenetically close strains were also reported in two *S. griseus* strains, which produced seven or four detectable strain-specific secondary metabolites [60]. By comparing the smBGCs sequences from the closely related strains, we revealed that ATCC 21113 could not produce chloramphenicol due to the single nucleotide deletion in the *cmlI* gene. In general, indel mutation is a major source of genomic variation with single nucleotide polymorphisms (SNPs) and is believed to be caused by DNA replication and repair error [61]. Interestingly, even after the mutation was reverse engineered to the sequence of other stains, the chloramphenicol production of ATCC 21113_NF was much lower than that of ATCC 10712 and ATCC 10595. This result indicates the higher repression of chloramphenicol production in ATCC 21113 than in the other strains. ATCC 10595 also produces a lower amount of chloramphenicol than ATCC 10712. By comparing the chloramphenicol BGCs from the three strains, we found several SNPs and indel mutations in genes, and different length of the cutoRNAs coding region between *cmlR* and *cmlLN* operon which are suspected as the main causes of the lower production level of chloramphenicol. In addition, a different number of specific tandem repeats in the upstream regions of the genes was found, which also affect the binding affinities of transcription factors for the regulation of chloramphenicol BGC.

ATCC 21113 was newly identified as a high producer of jadomycin among the strains, with a repressed chloramphenicol production. Considering the tendency that jadomycin production is high in low chloramphenicol producing strains and in the ethanol treatment condition, jadomycin and chloramphenicol production were regulated in an antagonistic manner. Meanwhile, among the jadomycin BGC sequence of the three strains (ATCC 10712, ATCC 10595, and ATCC 21113), many variations were found at the binding sites of two smBGC situated regulators, JadR2 and JadR*. Although further studies are needed to prove the effectiveness of these sequence variations on chloramphenicol and jadomycin production, the comparison of intraspecies variation helps to increase the secondary metabolite production by suggesting the novel engineering targets. Indeed, the increased clavulanic acid production was achieved by reverse engineering of a deletion mutation in the high clavulanic acid-producing *S. clavuligerus* strains, and such an engineering target was proposed by genome comparison between *S. clavuligerus* wild type strain and high-producing strains [62].

In conclusion, the high-quality genomes of three closely related *S. venezuelae* strains will serve as valuable resources for understanding the complicated secondary metabolism, and the sequence differences between the strains would help to design a strain capable of high production of valuable secondary metabolites.

## Figures and Tables

**Table 1:** Characteristics of the complete genomes of *S. venezuelae* strains.

Strain	ATCC 10712	ATCC 10595	ATCC 21113
Complete genome size (bp)	8,223,505	7,871,480	7,893,803
G + C content (%)	72.5	72.5	72.5
CDS	7377	6942	6987
rRNAs	21	21	21
tRNAs	67	67	67
Origin	Venezuela: Caracas, soil	USA: Illinois, compost soil	Unknown
Predicted secondary metabolite biosynthetic gene clusters	30	27	30
Accession No.	CP029197	CP029195	CP029196

## Supplementary Materials

The following are available online at https://www.mdpi.com/2218-273X/10/6/864/s1, **Figure S1**: Examples of corrected gene annotations from the jadomycin biosynthetic gene cluster (BGC), **Figure S2**: Phylogenetic analysis of *Streptomyces* species and *S. venezuelae* strains, **Figure S3**: Comparative genome analysis, **Figure S4:** Confirmation of loss or acquisition of secondary metabolite biosynthetic gene cluster (smBGC), **Figure S5**: Comparison of the chloramphenicol BGC of the three *S. venezuelae* strains, **Figure S6**: Comparison of the jadomycin BGC of the three *S. venezuelae* strains, **Table S1**: The isolated *Streptomyces* *venezuelae* strains, **Table S2**: The sequence-specific primers for detection of chloramphenicol BGC, **Table S3**: The sequences of oligos used for *S. venezuelae* ATCC 21113 engineering, **Table S4**: The mapping result of FR845719 fragments, separated by the position of ‘N’s, to the newly completed genome (CP029197), **Table S5**: Predicted smBGCs of the five *S. venezuelae* strains by antiSMASH, **Table S6**: Comparative analysis of smBGCs of *S. venezuelae* strains, **Table S7**: Protein sequence variations of chloramphenicol biosynthetic genes among the three *S. venezuelae* strains, **Table S8**: Protein sequence variations of jadomycin biosynthetic genes among the three *S. venezuelae* strains.
