# Supplementary material for: Comparative Genomics Determines Strain-Dependent Secondary Metabolite Production in Streptomyces venezuelae Strains
Source: Biomolecules. 2020 Jun 5;10(6):864. doi: 10.3390/biom10060864 (PMC7357120; doi:10.3390/biom10060864)
Supplement: Supplementary file 1 [file biomolecules-10-00864-s001.pdf]

Supplementary Information on

Comparative genomics determines strain-dependent secondary metabolite production in *Streptomyces venezuelae* strains

Woori Kim [1], Namil Lee [1], Soonkyu Hwang [1], Yongjae Lee [1], Jihun Kim [1], Suhyung Cho [1], Bernhard Palsson [2,3,4] and Byung-Kwan Cho [1,4,5,*]

[1] Department of Biological Sciences and KI for the BioCentury, Korea Advanced Institute of Science and Technology, Daejeon 34141, Korea; alfodnfl@kaist.ac.kr (W.K.); pig948@kaist.ac.kr (N.L.); geager@kaist.ac.kr (S.H.); ahrk7247@kaist.ac.kr (Y.L.); kjhwlgns0997@kaist.ac.kr (J.K.); shcho95@kaist.ac.kr (S.C.)
[2] Department of Bioengineering, University of California San Diego, La Jolla, CA 92093, USA; palsson@ucsd.edu (B.P.)
[3] Department of Pediatrics, University of California San Diego, La Jolla, CA 92093, USA
[4] Novo Nordisk Foundation Center for Biosustainability, Technical University of Denmark, Lyngby 2800, Denmark
[5] Intelligent Synthetic Biology Center, Daejeon 34141, Korea
* Correspondence: bcho@kaist.ac.kr; Tel.: +82-42-350-2660 (B.-K.C.)

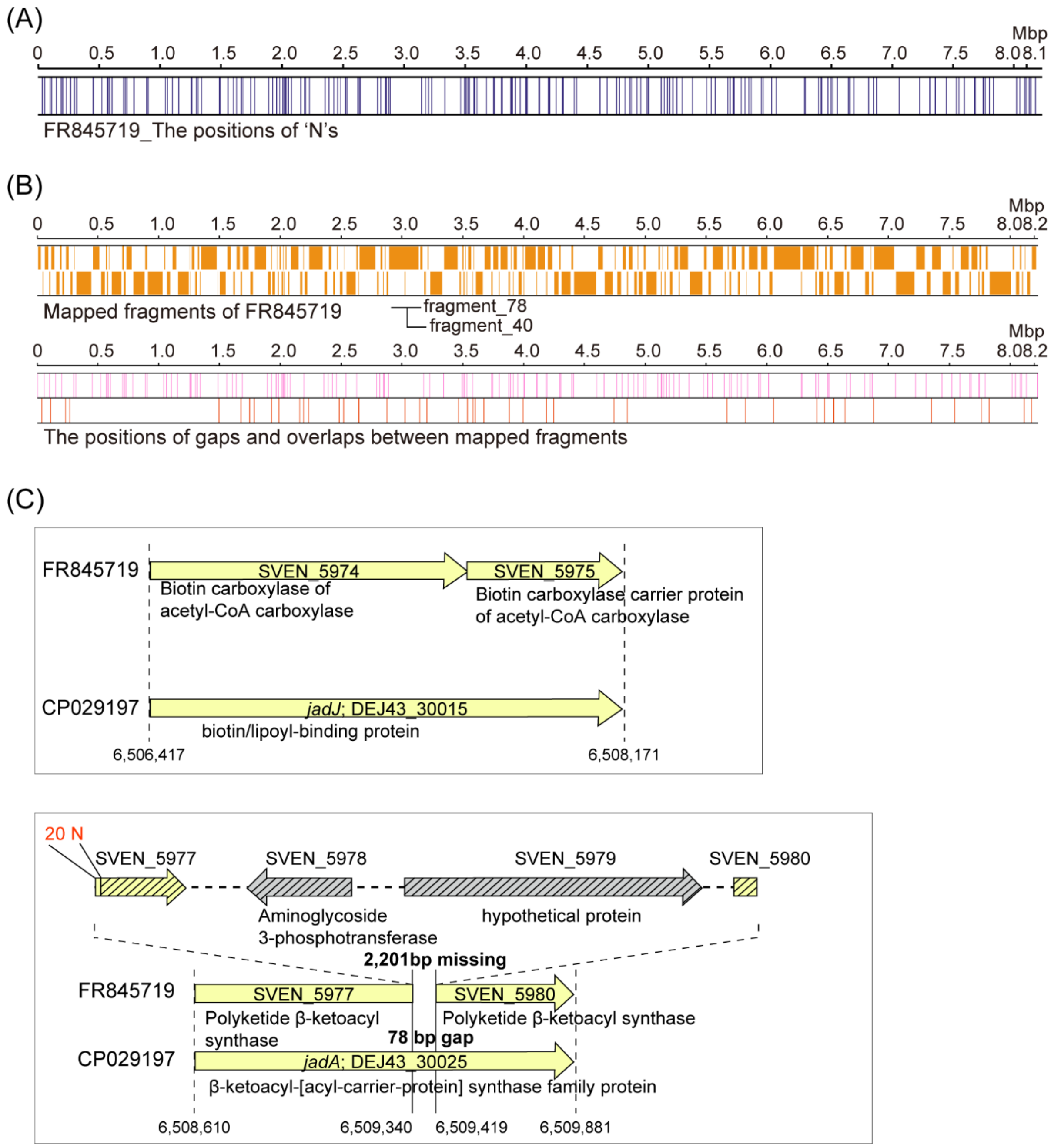

Figure S1. Examples of corrected gene annotations from the jadomycin biosynthetic gene cluster (BGC). (A) The positions of 'N's in the previously reported genome (FR845719). (B) The mapping of the FR845719 fragments, separated by the position of 'N's, to the newly completed genome (CP029197) and the positions of gaps and overlaps between the fragments. (C) The changed gene annotation of *jadJ* (upper) and *jadA* (bottom).

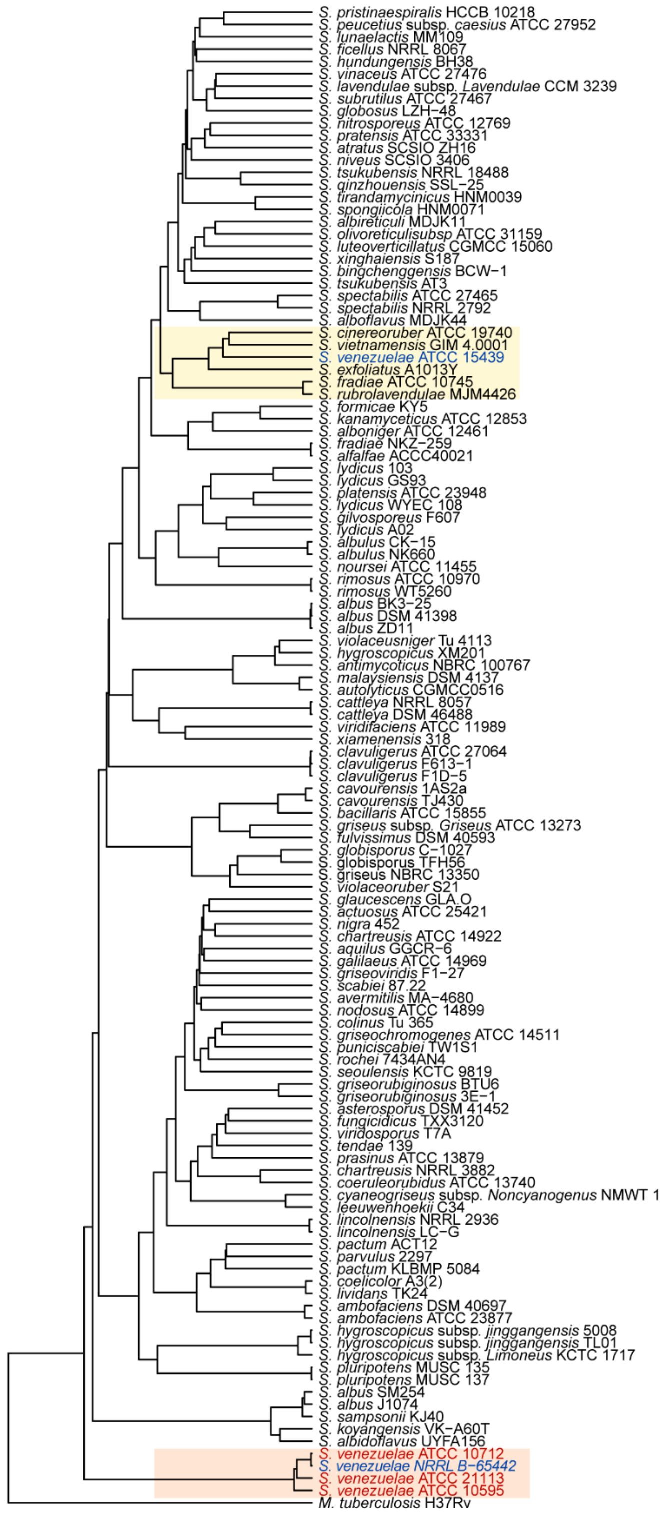

Figure S2. Phylogenetic analysis of *Streptomyces* species and *S. venezuelae* strains. A total of 121 *Streptomyces* strains, including five *S. venezuelae* strains, and *Mycobacterium tuberculosis* H37Rv were clustered according to the average nucleotide identity based on MUMmer algorithm (ANIm) values. The four closely related *S. venezuelae* strains were represented in the orange box. The strains that were clustered with *S. venezuelae* ATCC 15439 were located in the yellow box. In particular, three *S. venezuelae* strains of which genome sequence was completed in this study were colored as red and the other *S. venezuelae* strains were colored as blue.

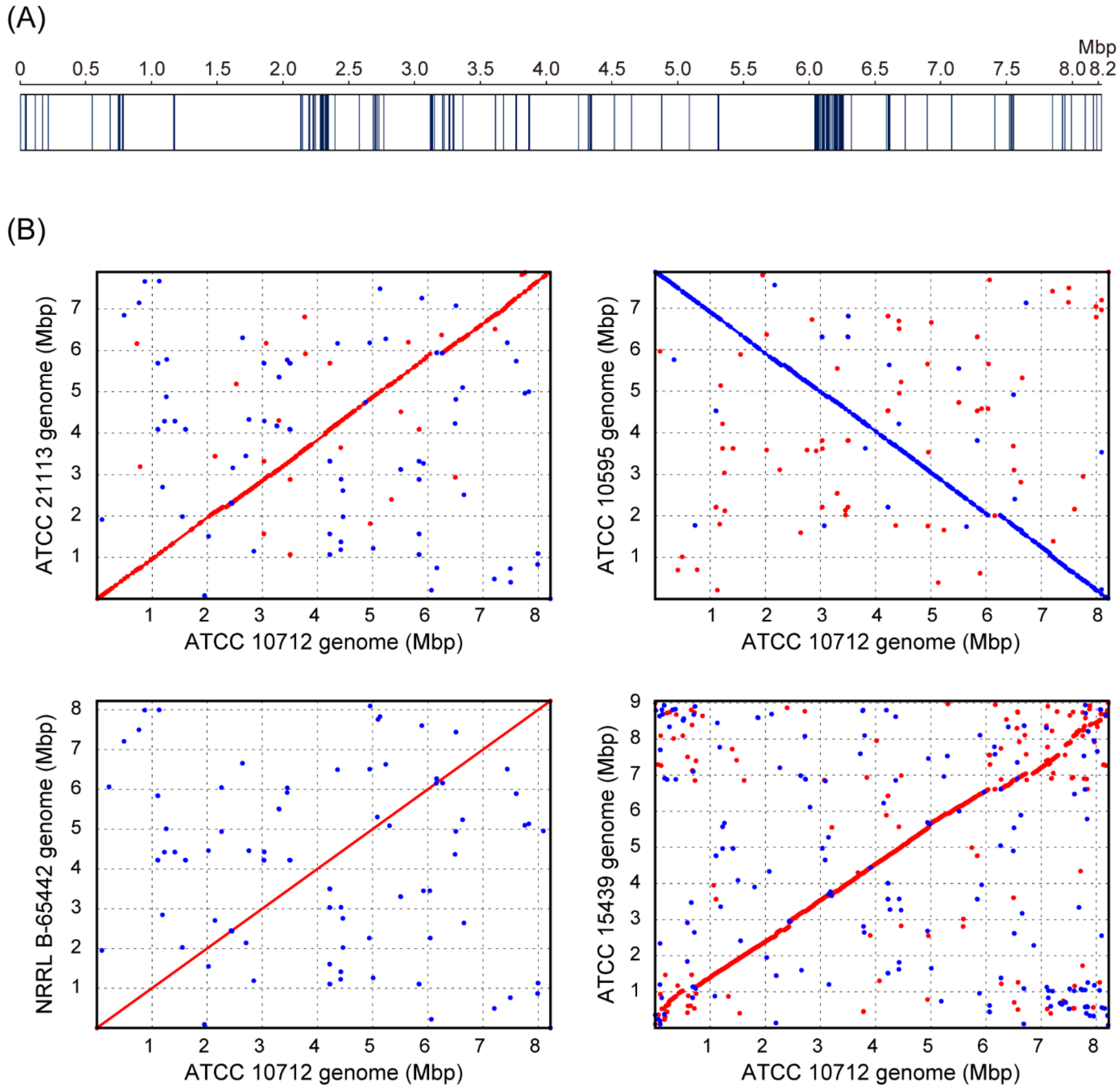

Figure S3. Comparative genome analysis. (A) The positions of orthologous genes between *S. venezuelae* ATCC 10712 and NRRL B-65442 at the genome of ATCC 10712. (B) Whole-genome alignments using nucmer.

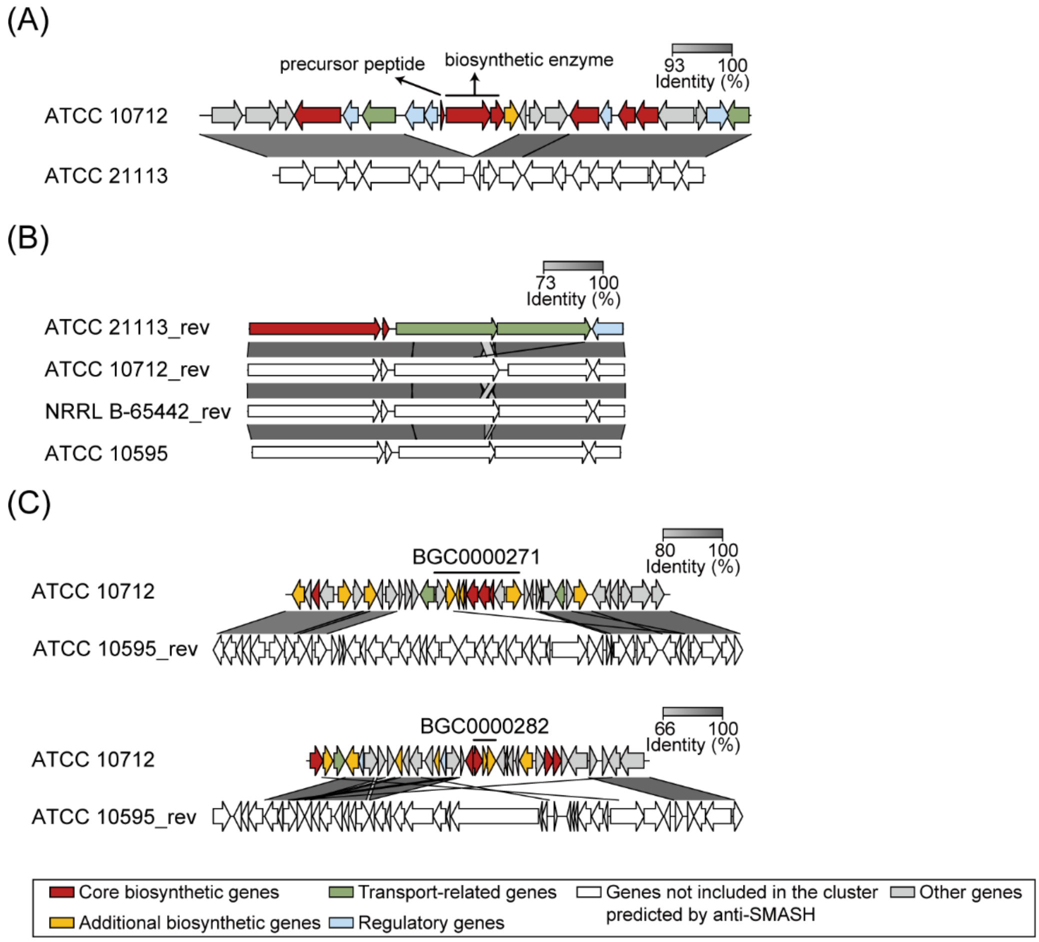

Figure S4. Confirmation of loss or acquisition of secondary metabolite biosynthetic gene cluster (smBGC). (A) Comparison of genomic regions, corresponding to lasso peptide BGC of ATCC 10712, between ATCC 21113 and ATCC 10712. (B) Finding of the lanthipeptide BGC of *S. venezuelae* ATCC 21113 from other *S. venezuelae* strains. (C) Comparison of genomic regions, corresponding to spore pigment BGC (Region No. 25) and alkylresorcinol BGC (Region No. 29) of ATCC 10712, between ATCC 10595 and ATCC 10712. The known spore pigment BGC and alkylresorcinol BGC are BGC0000271 and BGC0000282, respectively. The genes were colored by antiSMASH prediction, and the genes that are not included in the cluster predicted by antiSMASH were shown in white.

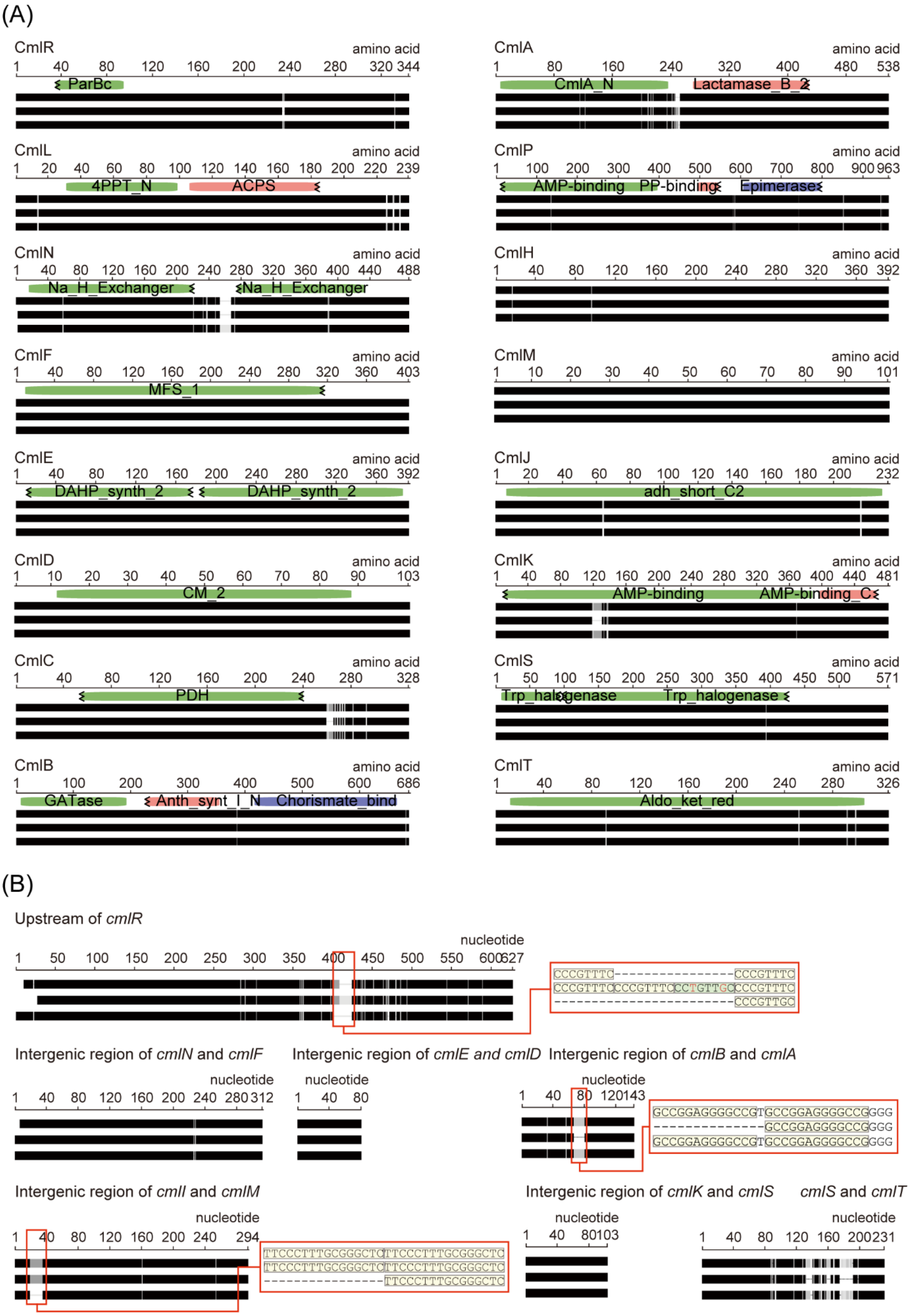

(A)
CmlR
amino acid
ParBc
CmlA
amino acid
CmlA_N
Lactamase_B_2
CmlL
amino acid
4PPT_N
ACPS
CmlP
amino acid
AMP-binding
PP-binding
Epimerase
CmlN
amino acid
Na_H_Exchanger
Na_H_Exchanger
CmlH
amino acid
CmlF
amino acid
MFS_1
CmlM
amino acid
CmlE
amino acid
DAHP_synth_2
DAHP_synth_2
CmlJ
amino acid
adh_short_C2
CmlD
amino acid
CM_2
CmlK
amino acid
AMP-binding
AMP-binding_C
CmlC
amino acid
PDH
CmlS
amino acid
Trp_halogenase
Trp_halogenase
CmlB
amino acid
GATase
Anth_synt_I_N
Chorismate_bind
CmlT
amino acid
Aldo_ket_red
(B)
Upstream of cmlR
nucleotide
CCCGTTTC----------------CCCGTTTC
CCCGTTTCCCCGTTTCCCTGTTGCCCCGTTTC
------------------------CCCGTTGC
Intergenic region of cmlN and cmlF
nucleotide
Intergenic region of cmlE and cmlD
nucleotide
Intergenic region of cmlB and cmlA
nucleotide
GCCGGAGGGGCCGTGCCGGAGGGGCCGGGG
--------------GCCGGAGGGGCCGGGG
GCCGGAGGGGCCGTGCCGGAGGGGCCGGGG
Intergenic region of cmlI and cmlM
nucleotide
TTCCCTTTGCGGGCTCTTCCCTTTGCGGGCTC
TTCCCTTTGCGGGCTCTTCCCTTTGCGGGCTC
----------------TTCCCTTTGCGGGCTC
Intergenic region of cmlK and cmlS
nucleotide
cmlS and cmlT
nucleotide

Figure S5. Comparison of the chloramphenicol BGC of the three *S. venezuelae* strains. (A) Comparison of the protein sequences of the genes in chloramphenicol BGC. The protein domain families were predicted using Pfam [1] and represented as green, pink, or purple color. The protein sequences were listed in the order of ATCC 10712, ATCC 21113, and ATCC 10595. The aligned protein sequences were depicted as black rectangles and the sequence variations were colored with gray. Blanks represent the deleted sequences. (B) Comparison of the intergenic regions of chloramphenicol BGC. The aligned nucleotide sequences were represented as black rectangles in the order of ATCC 10712, ATCC 21113, and ATCC 10595. Gray-colored regions represent the sequence variations among the strains and blanks are the position of deleted sequences. Repeat sequences were shown in the yellow box. The sequences were aligned using Geneious Prime 2020.0.5 (https://www.geneious.com).

(A)

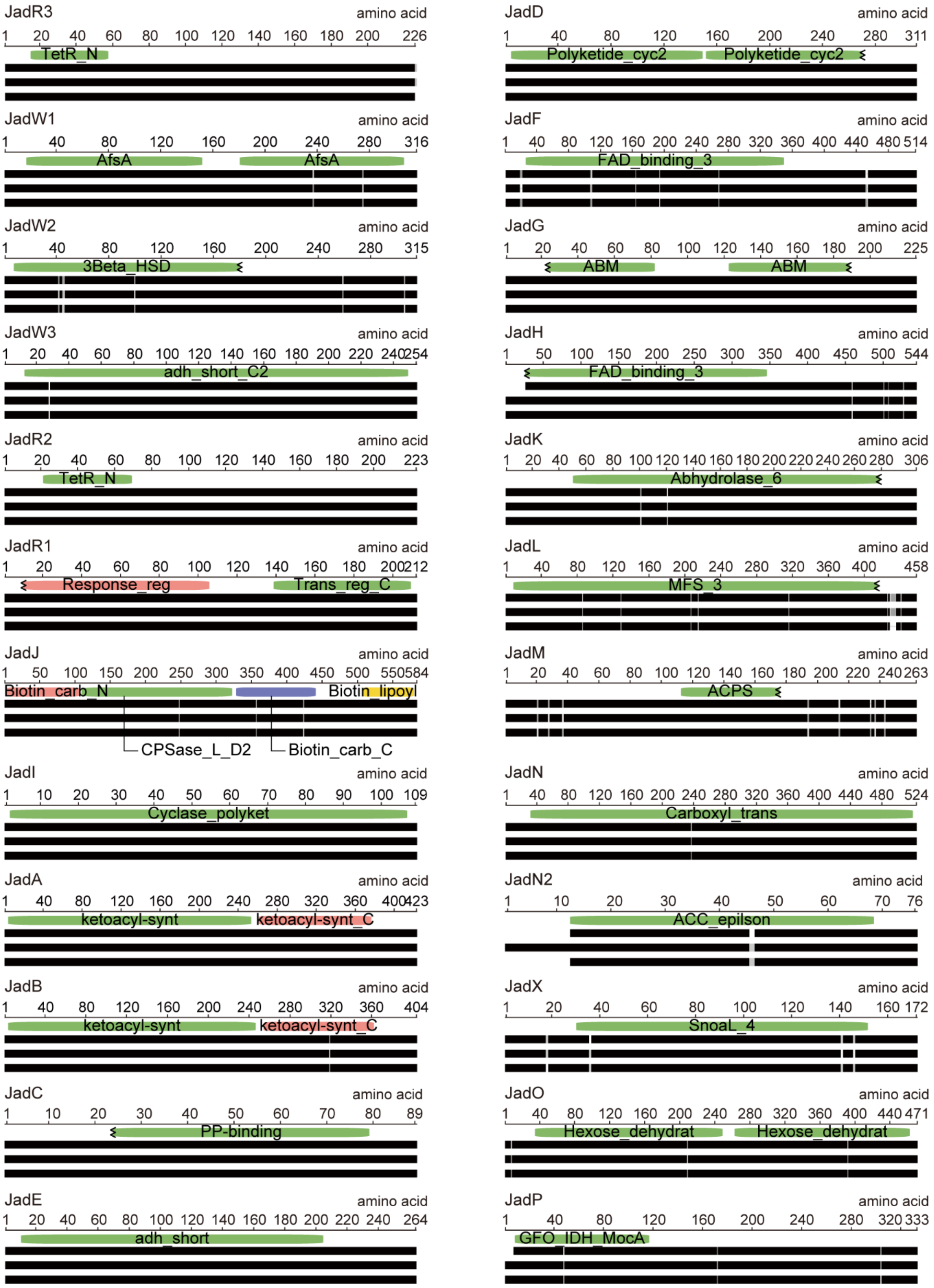
JadR3
amino acid
1 20 40 60 80 100 120 140 160 180 200 226
TetR_N
JadD
amino acid
1 40 80 120 160 200 240 280 311
Polyketide_cyc2
Polyketide_cyc2
JadW1
amino acid
1 40 80 120 160 200 240 280 316
AfsA
AfsA
JadF
amino acid
1 40 80 120 160 200 240 280 320 360 400 440 480 514
FAD_binding_3
JadW2
amino acid
1 40 80 120 160 200 240 280 315
3Beta_HSD
JadG
amino acid
1 20 40 60 80 100 120 140 160 180 200 225
ABM
ABM
JadW3
amino acid
1 20 40 60 80 100 120 140 160 180 200 220 240 254
adh_short_C2
JadH
amino acid
1 50 100 150 200 250 300 350 400 450 500 544
FAD_binding_3
JadR2
amino acid
1 20 40 60 80 100 120 140 160 180 200 223
TetR_N
JadK
amino acid
1 20 40 60 80 100 120 140 160 180 200 220 240 260 280 306
Abhydrolase_6
JadR1
amino acid
1 20 40 60 80 100 120 140 160 180 200 212
Response_reg
Trans_reg_C
JadL
amino acid
1 40 80 120 160 200 240 280 320 360 400 458
MFS_3
JadJ
amino acid
1 50 100 150 200 250 300 350 400 450 500 550 584
Biotin_carb_N
Biotin_lipoyl
CPSase_L_D2
Biotin_carb_C
JadM
amino acid
1 20 40 60 80 100 120 140 160 180 200 220 240 263
ACPS
JadI
amino acid
1 10 20 30 40 50 60 70 80 90 100 109
Cyclase_polyket
JadN
amino acid
1 40 80 120 160 200 240 280 320 360 400 440 480 524
Carboxyl_trans
JadA
amino acid
1 40 80 120 160 200 240 280 320 360 400 423
ketoacyl-synt
ketoacyl-synt_C
JadN2
amino acid
1 10 20 30 40 50 60 70 76
ACC_epilson
JadB
amino acid
1 40 80 120 160 200 240 280 320 360 404
ketoacyl-synt
ketoacyl-synt_C
JadX
amino acid
1 20 40 60 80 100 120 140 160 172
SnoaL_4
JadC
amino acid
1 10 20 30 40 50 60 70 80 89
PP-binding
JadO
amino acid
1 40 80 120 160 200 240 280 320 360 400 440 471
Hexose_dehydrat
Hexose_dehydrat
JadE
amino acid
1 20 40 60 80 100 120 140 160 180 200 220 240 264
adh_short
JadP
amino acid
1 40 80 120 160 200 240 280 320 333
GFO_IDH_MocA

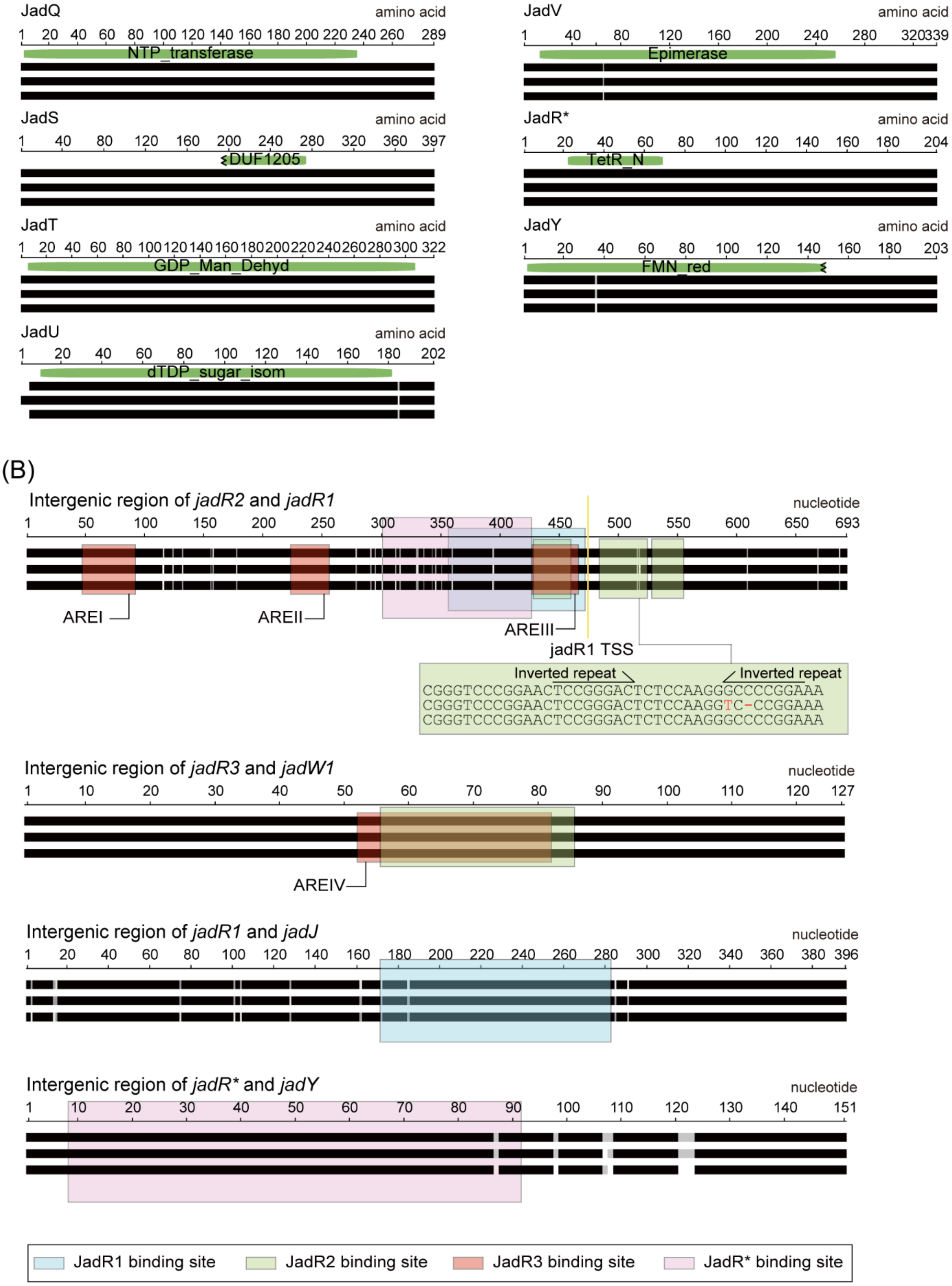
JadQ
amino acid
NTP_transferase
JadS
DUF1205
JadT
GDP_Man_Dehyd
JadU
dTDP_sugar_isom
JadV
Epimerase
JadR*
TetR_N
JadY
FMN_red
(B)
Intergenic region of jadR2 and jadR1
nucleotide
AREI
AREII
AREIII
jadR1 TSS
Inverted repeat
Inverted repeat
CGGGTCCCGGAACTCCGGGACTCTCCAAGGGCCCCGGAAA
CGGGTCCCGGAACTCCGGGACTCTCCAAGGTC-CCGGAAA
CGGGTCCCGGAACTCCGGGACTCTCCAAGGGCCCCGGAAA
Intergenic region of jadR3 and jadW1
AREIV
Intergenic region of jadR1 and jadJ
Intergenic region of jadR* and jadY
JadR1 binding site
JadR2 binding site
JadR3 binding site
JadR* binding site

Figure S6. Comparison of the jadomycin BGC of the three *S. venezuelae* strains. (A) Comparison of the protein sequences of jadomycin biosynthetic genes. The protein domain families were predicted using Pfam [1] and represented as green, pink, purple, or yellow color. The protein sequences from ATCC 10712, ATCC 21113, and ATCC 10595 were aligned and depicted as black rectangles. The sequence variations and the deleted positions were represented as gray color and blanks, respectively. (B) Comparison of the intergenic regions, which are binding sites of the regulators, of jadomycin BGC. The aligned nucleotide sequences were described as black rectangles and listed in the order of ATCC 10712, ATCC 21113, and ATCC 10595. The colored regions indicate the binding sites of the regulators. The sequences were aligned using Geneious Prime 2020.0.5 (https://www.geneious.com).

Table S1. The isolated *Streptomyces venezuelae* strains.

Strain	Origin	Product	Strain Designations	Chain of Custody
ATCC 10712 (Type strain)	Caracas, Venezuela; soil	chloramphenicol, jadomycin	04745, A-65, ATCC 25508, CBS 650.69, ETH 26169, IFO 13096, IMRU 3534, ISP 5230, KCC S-0526, NIHJ 38(0163), NRRL B-2277, RIA 1288, AS 4.1526, AS4.1307, BCRC 11512, CCRC 11512, CGMCC 4.1526, DSM 41109, IMET 41356, IMRU 3534, IMRU 3625, JCM 4526, Lanoot R-10801, LMG 19308, NBRC 12595, NBRC 13096, NIHJ 38 (0163), PD 04745, R-10801, R-8669, RIA 1288, RIA 70, VKM Ac-589	ATCC <-- Parke, Davis & Co., Ltd. <-- P. Burkholder A-65
ATCC 15439	Florence, Italy; soil	10-deoxymethynolide, methymycin	3627, M-2140, WC3627, BCRC 13602, CCRC 13602, CGMCC 4.1826, DSM 41110, IMRU 3627, M-2140	ATCC <-- SA Waksman <-- E.R. Squibb & Sons M-2140
ATCC 10595	Urbana, Illinois, USA; compost soil	chloramphenicol	8-44, Boots 274, BootsFD 274, IPV 877, NCIB 8231, NRRL B-902, PSA 135, RIA 130, RIA 741, BCRC 11510, CBS 369.49, CCRC 11510, CDBB 802, CECT 3113, Chas. Pfizer Co. FD-16317, DSM 40634, ETH 10210, FD 274, FDA 274, Gottlieb 8-44, IAW 123, ICMP 484, IPV 877, NCIM 2425, NCIMB 9008, VKM Ac-505	D Gottlieb
ATCC 15068	Oswego, New York, USA	methymycin	IMRU 3629, WC3629, BCRC 13461, CCRC 13461, DSM 41111	ATCC <-- IMRU-Institute of Mirobiology Rutgers Universitt <-- E.R. Squibb & Sons
ATCC 21113	.	xylose isomerase, glucose isomerase	BCRC 13603, CBS 691.73, CCRC 13603	CPC International, Inc.
ATCC 14585	; soil	anthelvencin, plasmid pVE1	A2640, BCRC 13604, CBS 764.70, CCRC 13604	Eli Lilly & Co.
ATCC 14584	; soil	anthelvencin	A2000, BCRC 13457, CBS 763.70, CCRC 13457	Eli Lilly & Co.
ATCC 14583	; soil	anthelvencin	A200, BCRC 13462, CBS 762.70, CCRC 13462	Eli Lilly & Co.
ATCC 21018	; soil	methionine decarboxylase	KY 591, K 67	ATCC <-- Kyowa Ferm. Ind. Co., Ltd. <-- K. Nakayama
ATCC 21782	Florida, USA; soil from Miami Beach	antibiotic EM-98	SC 7249, BCRC 13458, CCRC 13458	E.R. Squibb & Sons, Inc.
CECT 3069	San Martín de Valdeiglesias (Madrid), Spain; soil	lytic enzyme strepzyme RA	Rodriguez 11	CECT, 1978 <-- M.J. Rodriguez, Instituto de Biología Celular, CSIC, Madrid, Spain
CECT 7920	Leioa (Vizcaya), Spain; soil	.	.	CECT, 2011 <-- J.L. Serra, Univ. País Vasco, Spain

CGMCC 4.224	.	.	.	CGMCC <-- Institute of Medicinal Biotechnology CAMS A-189
CGMCC 4.244	.	.	.	CGMCC <-- Institute of Soil Science CAS 土 1
CGMCC 4.328	.	.	.	CGMCC <-- Institute of Microbiology CAS 17-23
CGMCC 4.439	.	.	.	CGMCC <-- Institute of Microbiology CAS 8-11
CGMCC 4.440	.	.	.	CGMCC <-- Institute of Microbiology CAS 8-13
CGMCC 4.5874	.	.	.	CGMCC <-- Hebei University 174005
CGMCC 4.632	.	.	.	CGMCC <-- Institute of Medicinal Biotechnology CAMS A-308
CGMCC 4.644	.	.	.	CGMCC <-- Institute of Microbiology CAS 12
DSM 40612	.	.	C 448, CCM 3198, Glaxo Lab.C.488, JCM 4690, KCC S-0690	DSM <-- CCM <-- Glaxo Lab., C 448
DSM 40727	.	dirinamycin, rinamycin.	Tü 1102	DSM <-- H. Zähner, Tü 1102
HAMBI 917	.	.	.	HAMBI <-- W. Kurylowicz <-- NHIJ 12-1 AS4-145
ICMP 467	United Kingdom	.	.	.
ICMP 928	South Canterbury, New Zealand	.	.	T.R. Vernon
ICMP 231	United Kingdom	.	.	.
MTCC 4218	.	.	.	MTCC <-- India <-- Dr.S.K.Sen,Santiniketan–731235
NCIM 2215	.	chloramphenicol	PRL X-58	.
NRRL B-65442	.	chloramphenicol	.	NRRL <-- John Innes Centre, Norwich, UK

Table S2. The sequence-specific primers for detection of chloramphenicol BGC.

Gene	Function	Sequence (5' to 3')	PCR length (bp)
cmlB (SVEN_0920)	4-amino-4-deoxychorismate synthase	Forward: TAGCCGAGGTTGAACTCGAA	924
		Reverse: GCTTCTGATCGACAACTACGACT	
cmlP (SVEN_0922)	nonribosomal peptide synthase	Forward: CCGCAGAACTCCACCTTC	794
		Reverse: GAACTCCCTGACCTGTTGACC	
cmlH (SVEN_0923)	amidase	Forward: GACGGCGAGTACCTCGTCTT	791
		Reverse: GTTCCACCACTTGCTCCAAT	

Table S3. The sequences of oligos used for *S. venezuelae* ATCC 21113 engineering.

Oligo	Sequence (5' to 3')
protospacer_1	ACGCGCGAGTCCGGCAGCACGCGT
protospacer_2	AAACACGCGTGCTGCCGGACTCGC
Editing_F	ATATTCTAGACAGATGGGCCCTGATCTGCC
Editing_R	ATATTCTAGAACGCTGCTGAACGGACTCAC
SDM_F	GCAGCACGCGGTCGGAGAAGTC
SDM_R	ACTTCTCCGACCGCGTGCTGCC
NF_Check_F	CAGCGGGAGTAGGGGTACTG
NF_Check_R	TCTTCCGGGAAACCTCGTCC
Sanger_check	CGCGACGATCGACTCCCAGG

Table S4. The mapping result of FR845719 fragments, separated by the position of 'N', to the newly completed genome (CP029197).

Fragment	Mapping position		Left misassembly (bp)	Overlap (bp)	Gap (bp)	Right misassembly (bp)
Fragment_1	480	36794			480	
Fragment_2	36786	58122		9		
Fragment_3	58180	100948			57	
Fragment_4	100981	111820	282		32	
Fragment_5	111817	151316		4		
Fragment_6	151360	191030			43	
Fragment_7	191031	199185				
Fragment_8	199238	233104			52	193
Fragment_9	233002	266596		103		2
Fragment_10	266386	297657		211		589
Fragment_11	297661	317959			3	
Fragment_12	317971	454469			11	
Fragment_13	454708	520421			238	
Fragment_14	520471	564075			49	
Fragment_15	564103	576667			27	
Fragment_16	576757	585879			89	
Fragment_17	585928	604117			48	
Fragment_18	604153	702269			35	
Fragment_19	702370	714733			100	304
Fragment_20	714774	727927			40	
Fragment_21	727982	786804			54	
Fragment_22	786818	888923			13	
Fragment_23	888969	902758			45	
Fragment_24	902798	1038935			39	
Fragment_25	1038962	1060074			26	2
Fragment_26	1060130	1102785	2		55	
Fragment_27	1106552	1153769			3766	
Fragment_28	1153841	1257414	7		71	
Fragment_29	1257679	1262075			264	
Fragment_30	1262115	1264655			39	
Fragment_31	1264809	1299631	67		153	
Fragment_32	1299692	1315305			60	
Fragment_33	1315360	1340829			54	
Fragment_34	1341822	1342843			992	
Fragment_35	1342900	1487163			56	
Fragment_36	1487191	1488896			27	
Fragment_37	1488930	1494385			33	
Fragment_38	1494344	1559546		42		
Fragment_39	1559569	1602507			22	
Fragment_41	1607290	1635383			4782	
Fragment_42	1635404	1674781			20	380
Fragment_43	1674675	1684164		107		
Fragment_44	1684189	1746348			24	
Fragment_45	1746338	1781324		11		578
Fragment_46	1781197	1893039		128		
Fragment_47	1893090	1925151			50	
Fragment_48	1925166	1929425			14	
Fragment_49	1929253	1961995	668	173		
Fragment_50	1962044	1982441			48	301
Fragment_51	1982476	1986850			34	333
Fragment_52	1986662	2012426		189		
Fragment_53	2012468	2021809	113		41	364
Fragment_54	2022324	2026959			514	2928
Fragment_55	2030291	2037515	56		3331	
Fragment_56	2037550	2057018	2		34	

Fragment_57	2057023	2082690			4	
Fragment_58	2082737	2157074			46	
Fragment_59	2157068	2186941		7		
Fragment_60	2186732	2193768	252	210		
Fragment_61	2193799	2228528	414		30	
Fragment_62	2228487	2353467		42		
Fragment_63	2353509	2390046			41	
Fragment_64	2390085	2428894			38	
Fragment_65	2428912	2458446			17	
Fragment_66	2458521	2481543	374		74	
Fragment_67	2481321	2519660	7	223		2
Fragment_68	2519659	2533989		2		
Fragment_69	2534489	2624960			499	
Fragment_70	2624971	2637779			10	
Fragment_71	2637770	2644468		10		1
Fragment_72	2644455	2786465		14		
Fragment_73	2786493	2813323			27	
Fragment_74	2813350	2848594			26	10
Fragment_75	2848623	2853048	182		28	
Fragment_76	2853097	2872542			48	1
Fragment_77	2872279	2887345		264		
Fragment_78	2887370	3144030	3		24	1
Fragment_40	3023337	3025124		1788		
Fragment_79	3143878	3177037	194	153		
Fragment_80	3177425	3201230			387	
Fragment_81	3201112	3225003	2	119		68
Fragment_82	3225033	3337620			29	
Fragment_83	3337811	3465965			190	240
Fragment_84	3465800	3494033		166		
Fragment_85	3498341	3503797			4307	
Fragment_86	3504495	3513677			697	
Fragment_87	3513710	3530823			32	
Fragment_88	3530916	3537572			92	
Fragment_89	3537523	3575938	156	50		
Fragment_90	3575975	3580542			36	
Fragment_91	3580451	3602035	71	92		1
Fragment_92	3601829	3673329		207		
Fragment_93	3673143	3736341		187		
Fragment_94	3736394	3742995			52	
Fragment_95	3743029	3797895	471		33	296
Fragment_96	3797917	3800599			21	1
Fragment_97	3800723	3873893			123	
Fragment_98	3873948	3879620	365		54	
Fragment_99	3879469	3885705	545	152		
Fragment_100	3885721	3886878			15	
Fragment_101	3887108	3912268			229	
Fragment_102	3912299	3912982	215		30	
Fragment_103	3913362	3953414			379	
Fragment_104	3953493	3993329			78	679
Fragment_105	3993223	3999690		107		1
Fragment_106	3999728	4006012			37	229
Fragment_107	4006045	4105406			32	
Fragment_108	4105453	4107959			46	
Fragment_109	4108004	4180017	244		44	2
Fragment_110	4180065	4185211			47	
Fragment_111	4185208	4188432		4		95
Fragment_112	4188472	4190279			39	
Fragment_113	4190329	4245023			49	
Fragment_114	4245018	4295159		6		
Fragment_115	4295193	4302563			33	

Fragment_116	4302610	4394636			46	
Fragment_117	4394679	4409085	338		42	293
Fragment_118	4409122	4603149			36	
Fragment_119	4603213	4658103			63	6
Fragment_120	4658147	4738822	477		43	
Fragment_121	4738654	4763143		169		
Fragment_122	4763182	4810420			38	
Fragment_123	4810514	4848663			93	
Fragment_124	4848487	4856213		177		
Fragment_125	4856280	4904358			66	
Fragment_126	4904405	4937304			46	
Fragment_127	4937335	4939074			30	
Fragment_128	4939233	4986263	207		158	
Fragment_129	4986500	5002215			236	
Fragment_130	5002256	5099764			40	
Fragment_131	5100892	5128198			1127	
Fragment_132	5128221	5131344			22	
Fragment_133	5131357	5152534			12	
Fragment_134	5152539	5174216			4	
Fragment_135	5174258	5197837			41	
Fragment_136	5197922	5231997			84	
Fragment_137	5232076	5275773			78	
Fragment_138	5275816	5279320			42	
Fragment_139	5279356	5357899	124		35	
Fragment_140	5357909	5475763			9	
Fragment_141	5475858	5513345			94	
Fragment_142	5513461	5547262			115	
Fragment_143	5547313	5648950			50	
Fragment_144	5649010	5669434			59	
Fragment_145	5669244	5692224	3	191		
Fragment_146	5692290	5703646			65	
Fragment_147	5703704	5756335			57	
Fragment_148	5756365	5787999			29	
Fragment_149	5788006	5822809			6	
Fragment_150	5822662	5839738		148		
Fragment_151	5843594	5928680			3855	441
Fragment_152	5928731	5943332			50	
Fragment_153	5943511	6013507			178	
Fragment_154	6013562	6052489			54	730
Fragment_155	6052418	6280900		72		
Fragment_156	6280922	6286856			21	
Fragment_157	6286925	6399604			68	
Fragment_158	6399645	6409102			40	
Fragment_159	6408985	6422660	76	118		
Fragment_160	6422666	6470351			5	
Fragment_161	6470161	6497290	1	191		
Fragment_162	6497349	6509340			58	
Fragment_163	6509419	6547169	2174		78	1
Fragment_164	6547096	6638302		74		
Fragment_165	6638294	6652575		9		
Fragment_166	6652641	6685393			65	
Fragment_167	6685430	6801346	324		36	
Fragment_168	6801417	6845596			70	
Fragment_169	6845621	6865602			24	
Fragment_170	6865657	6871248			54	3
Fragment_171	6870971	7051916		278		
Fragment_172	7052021	7219679			104	1
Fragment_173	7219834	7305500			154	
Fragment_174	7305549	7352134			48	
Fragment_175	7351904	7438053	897	231		

Fragment_176	7438097	7519114	352		43	6
Fragment_177	7519146	7539697	1		31	
Fragment_178	7539599	7583324	264	99		
Fragment_179	7583376	7667159			51	
Fragment_180	7667209	7677813			49	
Fragment_181	7677968	7738647	1		154	
Fragment_182	7738696	7747527			48	
Fragment_183	7747579	7760159			51	
Fragment_184	7760144	7791244		16		
Fragment_185	7791349	7822750			104	
Fragment_186	7822722	8014244		29		
Fragment_187	8014282	8042624			37	
Fragment_188	8042642	8081299			17	
Fragment_189	8081353	8091163			53	
Fragment_190	8091165	8115193	413		1	
Fragment_191	8115003	8132746		191		
Fragment_192	8132785	8170224			38	1
Fragment_193	8170051	8222973		174		
					532	

Table S5. Predicted smBGCs of the five *S. venezuelae* strains by antiSMASH.

Strain	Region	Type	From	To	antiSMASH prediction		Reference-based	
					Most similar known cluster	Similarity	Known cluster	Reference
ATCC 10712	1	ectoine	237,663	248,079	Ectoine	100%		
	2	terpene	274,351	295,302	Geosmin	100%		
	3	T1PKS,NRPS-like, T3PKS,NRPS	503,449	603,058	Venemycin, Thiazostatin / watasemycin A / watasemycin B / 2-hydroxyphenylthiazoline enantiopyochelin / isopyochelin	100%, 100%	Venemycin, Watasemycin, Isopyochelin, Thiazostatin	[2,3]
	4	lanthipeptide,terpene	614,320	643,318	Chrysomycin	5%	Isodauc-8-en-11-ol	[4]
	5	lanthipeptide	706,670	729,522	Venezuelin	75%	Venezuelin	[5]
	6	indole	866,720	889,926	Rebeccamycin	22%		
	7	NRPS-like	1,030,635	1,072,739	Chloramphenicol	100%	Chloramphenicol	[6]
	8	other	2,061,609	2,101,187	Malacidin	5%		
	9	siderophore	2,799,249	2,810,183	Desferrioxamine B	100%		
	10	lassopeptide	3,411,369	3,433,728	Ikarugamycin	8%		
	11	NRPS-like	4,408,662	4,450,901	Lactonamycin	10%		
	12	butyrolactone	4,522,587	4,531,360	Scleric acid	23%	Gaburedin	[7]
	13	melanin	5,002,462	5,010,679	Istamycin	8%		
	14	other, butyrolactone	5,475,955	5,517,060	A-factor	100%		
	15	LAP, thiopeptide	5,526,152	5,559,470	BD-12	17%		
	16	T3PKS	5,784,770	5,823,048	Flaviolin	50%		
	17	siderophore	5,873,375	5,885,837	Murayaquinone	6%		
	18	siderophore	5,938,831	5,953,252	Ficellomycin	5%		
	19	bacteriocin	6,353,037	6,363,866				
	20	T2PKS, butyrolactone	6,474,034	6,545,829	Rabelomycin	33%	Jadomycin	[8]
	21	NRPS-like	6,674,266	6,714,825	Formicamycins A-M	18%		
	22	NRPS,ladderane	6,720,536	6,853,750	Skyllamycin, Ulleungmycin	24%, 27%		
	23	terpene	7,021,150	7,046,269	Hopene	69%		
	24	bacteriocin	7,127,836	7,138,690				
	25	T2PKS	7,404,285	7,476,797	Spore pigment	83%		
	26	melanin	7,482,644	7,493,033	Melanin	28%		
	27	NRPS	7,704,842	7,757,706	Salinichelins	61%	Foroxymithine	[9]
	28	terpene	7,786,268	7,806,390	2-methylisoborneol	100%		
	29	T3PKS	7,943,622	7,984,779	Alkylresorcinol	100%		
	30	terpene,NRPS	8,186,751	8,223,505				
NRRL B-65442	1	ectoine	237,110	247,526	Ectoine	100%		
	2	terpene	273,798	294,749	Geosmin	100%		
	3	T1PKS,NRPS-like, T3PKS,NRPS	502,896	602,505	Venemycin, Thiazostatin / watasemycin A / watasemycin B / 2-hydroxyphenylthiazoline enantiopyochelin / isopyochelin	100%, 100%		
	4	lanthipeptide,terpene	613,767	642,765	Chrysomycin	5%		
	5	lanthipeptide	706,117	728,969	Venezuelin	75%		
	6	indole	866,168	889,374	Rebeccamycin	22%		

	7	NRPS-like	1,030,083	1,072,187	Chloramphenicol	100%	Chloramphenicol	[10]
	8	CDPS	2,070,178	2,090,903	Malacidin	5%		
	9	siderophore	2,798,699	2,809,633	Desferrioxamine B	100%		
	10	lassopeptide	3,410,820	3,433,179	Ikarugamycin	8%		
	11	NRPS-like	4,408,114	4,450,353	Lactonamycin	10%		
	12	butyrolactone	4,522,040	4,530,221	Scleric acid	23%		
	13	melanin	5,001,915	5,010,131	Istamycin	8%		
	14	other,butyrolactone	5,475,407	5,516,513	A-factor	100%		
	15	LAP,thiopeptide	5,525,842	5,558,923	BD-12	17%		
	16	T3PKS	5,784,219	5,822,500	Flaviolin	50%		
	17	siderophore	5,872,827	5,885,289	Murayaquinone	6%		
	18	siderophore	5,938,343	5,952,704	Ficellomycin	3%		
	19	bacteriocin	6,352,489	6,363,318				
	20	T2PKS,butyrolactone	6,473,487	6,545,282	Auricin, Rabelomycin	55%, 33%		
	21	NRPS-like	6,673,724	6,714,283	Formicamycins A-M	18%		
	22	NRPS,ladderane	6,719,994	6,853,094	Ulleungmycin	30%		
	23	terpene	7,020,609	7,045,713	Hopene	69%		
	24	bacteriocin	7,127,295	7,138,149				
	25	T2PKS	7,403,744	7,476,256	Spore pigment	83%		
	26	melanin	7,482,101	7,492,490	Melanin	28%		
	27	NRPS	7,704,299	7,757,163	Salinichelins	61%		
	28	terpene	7,785,725	7,805,848	2-methylisoborneol	100%		
	29	T3PKS	7,943,145	7,984,236	Alkylresorcinol	100%		
	30	terpene,NRPS	8,186,208	8,222,198				
ATCC 21113	1	ectoine	226,340	236,756	Ectoine	100%		
	2	terpene	262,951	283,790	Geosmin	100%		
	3	T1PKS,NRPS-like, T3PKS,NRPS	491,933	587,008	Venemycin, Thiazostatin / watasemycin A / watasemycin B / 2-hydroxyphenylthiazoline enantiopyochelin / isopyochelin	100%, 100%		
	4	lanthipeptide,terpene	598,226	627,153	Chrysomycin	5%		
	5	lanthipeptide	690,347	711,972	Venezuelin	75%		
	6	indole	830,483	853,689	Rebeccamycin	25%		
	7	NRPS-like	996,554	1,038,238	Chloramphenicol	100%		
	8	other	2,025,610	2,065,607	Malacidin	5%		
	9	siderophore	2,656,760	2,666,158	Desferrioxamine B	100%		
	10	NRPS-like	4,279,198	4,321,577	Lactonamycin	10%		
	11	butyrolactone	4,392,670	4,400,819	Scleric acid	17%		
	12	melanin	4,876,666	4,884,879	Istamycin	8%		
	13	other,butyrolactone	5,325,996	5,367,102	A-factor	100%		
	14	LAP,thiopeptide	5,376,311	5,409,422	BD-12	17%		
	15	T3PKS	5,634,832	5,673,000	Flaviolin	50%		
	16	siderophore	5,723,207	5,735,640	Murayaquinone	6%		
	17	siderophore	5,787,919	5,802,418	Ficellomycin	5%		
	18	bacteriocin	6,026,832	6,037,506				
	19	T2PKS,butyrolactone	6,149,363	6,221,464	Rabelomycin	33%		
	20	NRPS-like	6,323,470	6,364,558	Formicamycins A-M	18%		
	21	NRPS,ladderane	6,369,956	6,502,722	Ulleungmycin	30%		

	22	terpene	6,667,279	6,692,461	Hopene	69%		
	23	lanthipeptide	6,708,548	6,731,175	SAL-2242	100%		
	24	bacteriocin	6,775,670	6,786,524				
	25	T2PKS	7,044,639	7,117,151	Spore pigment	83%		
	26	melanin	7,135,800	7,146,189	Melanin	28%		
	27	NRPS	7,364,703	7,417,779	Salinichelins	61%		
	28	terpene	7,443,959	7,464,115	2-methylisoborneol	100%		
	29	T3PKS	7,620,391	7,661,548	Alkylresorcinol	100%		
	30	terpene,NRPS	7,857,641	7,893,803	A54145	3%		
	1	NRPS,terpene	372	42,904	A54145	3%		
	2	terpene	417,683	437,704	Ebelactone	5%		
	3	NRPS	455,436	508,016	Salinichelins	61%		
	4	melanin	707,970	717,404	Melanin	28%		
	5	bacteriocin	1,119,839	1,128,807				
	6	terpene	1,210,261	1,235,579	Hopene	69%		
	7	NRPS,ladderane, NRPS-like	1,401,624	1,576,477	Ulleungmycin, Formicamycins A-M	30%, 18%		
	8	T2PKS,butyrolactone	1,715,997	1,788,479	Rabelomycin	33%		
	9	bacteriocin	1,907,894	1,919,294				
	10	siderophore	2,100,914	2,112,875	Ficellomycin	5%		
	11	siderophore	2,166,808	2,179,325	Murayaquinone	6%		
	12	T3PKS	2,229,542	2,267,759	Flaviolin	50%		
	13	thiopeptide,LAP	2,488,993	2,522,342	BD-12	17%		
	14	other,butyrolactone	2,533,411	2,572,501	A-factor	100%		
	15	melanin	3,029,148	3,039,558	Istamycin	8%		
ATCC 10595	16	butyrolactone	3,515,505	3,526,542	Scleric acid	17%		
	17	NRPS-like	3,594,210	3,636,332	Lactonamycin	10%		
	18	lassopeptide	4,596,214	4,618,573	Ikarugamycin	8%		
	19	siderophore	5,170,979	5,182,757	Desferrioxamine B	100%		
	20	other	5,808,175	5,848,900	Malacidin	5%		
	21	NRPS-like	6,834,370	6,876,679	Chloramphenicol	100%	Chloramphenicol	[11]
	22	indole	7,018,119	7,041,325	Rebeccamycin	22%		
	23	lanthipeptide	7,173,282	7,195,122	Venezuelin	75%		
	24	terpene,lanthipeptide	7,255,794	7,285,276	Chrysomycin	5%		
	25	NRPS,T3PKS, T1PKS,NRPS-like	7,293,769	7,392,081	Venemycin, Thiazostatin / watasemycin A / watasemycin B / 2-hydroxyphenylthiazoline enantiopyochelin / isopyochelin	100%, 100%		
	26	terpene	7,603,978	7,626,164	Geosmin	100%		
	27	ectoine	7,653,128	7,663,544	Ectoine	100%		
	1	NRPS-like	235,450	277,555				
	2	terpene	566,626	586,665	2-methylisoborneol	100%		
	3	ectoine	638,778	649,197	Ectoine	100%		
	4	T2PKS,terpene	671,615	742,012	Spore pigment	83%		
ATCC 15439	5	terpene	764,885	786,181	Geosmin	100%		
	6	NRPS,bacteriocin	1,022,749	1,090,710	Salinichelins	61%		
	7	NRPS,lanthipeptide	1,101,677	1,150,815	Venezuelin	100%		
	8	T3PKS	1,157,806	1,198,963	Alkylresorcinol	100%		
	9	indole	1,270,190	1,293,429	Rebeccamycin	36%		

10	other	2,447,944	2,487,869	Malacidin	5%		
11	PKS-like,T1PKS,T2PKS	2,550,460	2,655,796	Aranciamycin	34%		
12	lanthipeptide	2,865,209	2,889,945	Kanamycin	3%		
13	siderophore	3,314,571	3,324,611	Desferrioxamine B	100%		
14	thiopeptide,LAP	3,830,914	3,861,094				
15	NRPS	4,300,066	4,351,419	Dutomycin	4%		
16	cyanobactin	4,935,747	4,958,113				
17	NRPS-like	4,958,353	5,000,972	Xantholipin, Lactonamycin	10%, 8%		
18	T1PKS	5,571,781	5,644,226	Rosamicin / salinipyrone A / pacificanone A	60%	Pikromycin	[12]
19	melanin	5,666,875	5,675,070	Istamycin	8%		
20	other	6,094,283	6,135,389	Stambomycin	16%		
21	siderophore	6,505,738	6,520,209	Ficellomycin	5%		
22	bacteriocin	6,700,487	6,710,921				
23	butyrolactone	6,859,728	6,870,678	Griseoviridin / viridogrisein	8%		
24	NRPS,T1PKS	6,988,565	7,034,327	Salinosporamide A, Formicamycins A-M	23%, 18%		
25	terpene	7,230,181	7,256,061	Hopene	69%		
26	bacteriocin	7,568,838	7,580,283				
27	lanthipeptide,melanin	7,874,236	7,896,625	Melanin	28%		
28	NRPS,T1PKS, lanthipeptide	7,903,849	7,963,413	Oxazolomycin	21%		
29	NRPS,T1PKS	7,971,806	8,089,981	Streptolydigin	21%		
30	betalactone,NRPS-like	8,347,253	8,391,161	Kanamycin	4%		
31	terpene	8,781,404	8,802,300				

Table S6. Comparative analysis of smBGCs of *S. venezuelae* strains.

Type	ATCC 10712		NRRL B-65442		ATCC 21113		ATCC 10595		Conservation analysis of *S. venezuelae* strains	ATCC 15439			Conservation analysis of ATCC 15439 strain
	Region No.	Most similar known cluster	Region No.	Most similar known cluster	Region No.	Most similar known cluster	Region No.	Most similar known cluster		Region No.	Type	Most similar known cluster	
										1	NRPS-like	N.M.	15439-specific
Ectoine	1	Ectoine (100%)	1	Ectoine (100%)	1	Ectoine (100%)	27	Ectoine (100%)	Conserved	3	Ectoine	Ectoine (100%)	Conserved
Terpene	2	Geosmin (100%)	2	Geosmin (100%)	2	Geosmin (100%)	26	Geosmin (100%)	Conserved	5	Terpene	Geosmin (100%)	Conserved
T1PKS,NRPS-like,T3PKS, NRPS	3	Venemycin, Thiazostatin, Watasemycin, Isopyochelin (100%)	3	Venemycin, Thiazostatin, Watasemycin, Isopyochelin (100%)	3	Venemycin, Thiazostatin, Watasemycin, Isopyochelin (100%)	25	Venemycin, Thiazostatin, Watasemycin, Isopyochelin (100%)	Conserved				Missing
Lanthipeptide, Terpene	4	Chrysomycin (5%) known as Isodauc-8-en-11-ol	4	Chrysomycin (5%)	4	Chrysomycin (5%)	24	Chrysomycin (5%)	Conserved				Missing
Lanthipeptide	5	Venezuelin (75%)	5	Venezuelin (75%)	5	Venezuelin (75%)	23	Venezuelin (75%)	Conserved	7	NRPS, Lanthipeptide	Venezuelin (100%)	Conserved, 15439-specific: NRPS
Indole	6	Rebeccamycin (22%)	6	Rebeccamycin (22%)	6	Rebeccamycin (25%)	22	Rebeccamycin (22%)	Conserved	9	Indole	Rebeccamycin (36%)	Conserved
NRPS-like	7	Chloramphenicol (100%)	7	Chloramphenicol (100%)	7	Chloramphenicol (100%)	21	Chloramphenicol (100%)	Conserved				Missing
Other	8	Malacidin (5%)	8	Malacidin (5%)	8	Malacidin (5%)	20	Malacidin (5%)	Conserved	10	Other	Malacidin (5%)	Conserved
										11	PKS-like, T1PKS, T2PKS	Aranciamycin (34%)	15439-specific
										12	Lanthipeptide	Kanamycin (3%)	15439-specific
Siderophore	9	Desferrioxamine B (100%)	9	Desferrioxamine B (100%)	9	Desferrioxamine B (100%)	19	Desferrioxamine B (100%)	Conserved	13	Siderophore	Desferrioxamin B (100%)	Conserved
Lassopeptide	10	Ikarugamycin (8%)	10	Ikarugamycin (8%)			18	Ikarugamycin (8%)	Conserved, ATCC 21113 -missing				Missing
										14	Thiopeptide, LAP	N.M.	15439-specific
										15	NRPS	Dutomycin (4%)	15439-specific

										16	Cyanobactin		15439-specific
NRPS-like	11	Lactonamycin (10%)	11	Lactonamycin (10%)	10	Lactonamycin (10%)	17	Lactonamycin (10%)	Conserved	17	NRPS-like	Xantholipin (10%), Lactonamycin (8%)	Conserved
Butyrolactone	12	Scleric acid (23%) known as Gaburedin	12	Scleric acid (23%)	11	Scleric acid (17%)	16	Scleric acid (17%)	Conserved				Missing
										18	T1PKS	Rosamicin/ Salinipyrone A/ Pacificanone A (60%) known as Pikromycin	15439-specific
Melanin	13	Istamycin (8%)	13	Istamycin (8%)	12	Istamycin (8%)	15	Istamycin (8%)	Conserved	19	Melanin	Istamycin (8%)	Conserved
Other, Butyrolactone	14	A-factor (100%)	14	A-factor (100%)	13	A-factor (100%)	14	A-factor (100%)	Conserved	20	Other	Stambomycin (16%)	Partially conserved
LAP, Thiopeptide	15	BD-12 (17%)	15	BD-12 (17%)	14	BD-12 (17%)	13	BD-12 (17%)	Conserved				Missing
T3PKS	16	Flaviolin (50%)	16	Flaviolin (50%)	15	Flaviolin (50%)	12	Flaviolin (50%)	Conserved				Missing
Siderophore	17	Murayaquinone (6%)	17	Murayaquinone (6%)	16	Murayaquinone (6%)	11	Murayaquinone (6%)	Conserved				Missing
Siderophore	18	Ficellomycin (5%)	18	Ficellomycin (3%)	17	Ficellomycin (5%)	10	Ficellomycin (5%)	Conserved	21	Siderophore	Ficellomycin (5%)	Conserved
Bacteriocin	19	N.M.	19	N.M.	18	N.M.	9	N.M.	Conserved	22	Bacteriocin	N.M.	Conserved
T2PKS, Butyrolactone	20	Rabelomycin (33%) known as Jadomycin	20	Auricin (55%), Rabelomycin (33%)	19	Rabelomycin (33%)	8	Rabelomycin (33%)	Conserved	23	Butyrolactone	Griseoviridin/viridogrisein (8%)	Partially conserved
NRPS-like	21	Formicamycins A-M (18%)	21	Formicamycins A-M (18%)	20	Formicamycins A-M (18%)	7	Ulleungmycin (30%), Formicamycins A-M (18%)	Conserved	24	NRPS, T1PKS	salinosporamide A (23%), Formicamycins A-M (18%)	Partially conserved, 15439-specific: NRPS, T1PKS
NRPS, Ladderane	22	Skyllamycin (24%), Ulleungmycin (27%)	22	Ulleungmycin (30%)	21	Ulleungmycin (30%)			Conserved				Missing
Terpene	23	Hopene (69%)	23	Hopene (69%)	22	Hopene (69%)	6	Hopene (69%)	Conserved	25	Terpene	Hopene (69%)	Conserved

Lanthipeptide		DEJ43_32430 - DEJ43_32520, core biosynthetic genes: DEJ43_32480		vnz_RS32230 - vnz_RS32320, core biosynthetic genes: vnz_RS32280	23	SAL-2242 (100%)		DEJ45_04990 - DEJ43_05070, core biosynthetic genes: DEJ45_05020	Conserved				Missing
Bacteriocin	24	N.M.	24	N.M.	24	N.M.	5	N.M.	Conserved				Missing
										26	Bacteriocin	N.M.	15439-specific
T2PKS	25	Spore pigment (83%)	25	Spore pigment (83%)	25	Spore pigment (83%)			Conserved, ATCC 10595 -missing	4	T2PKS, Terpene	Spore pigment (83%)	Conserved in other regions, 15439-specific: Terpene
Melanin	26	Melanin (28%)	26	Melanin (28%)	26	Melanin (28%)	4	Melanin (28%)	Conserved	27	Lanthipeptide, Melanin	Melanin (28%)	Conserved, 15439-specific: Lanthipeptide
NRPS	27	Salinichelins (61%) known as Foroxymithine	27	Salinichelins (61%)	27	Salinichelins (61%)	3	Salinichelins (61%)	Conserved	6	NRPS, Bacteriocin	Salinichelins (61%)	Conserved in other regions, 15439-specific: Bacteriocin
Terpene	28	2-methylisoborneol (100%)	28	2-methylisoborneol (100%)	28	2-methylisoborneol (100%)	2	2-methylisoborneol (100%)	Conserved	2	Terpene	2-methylisoborneol (100%)	Conserved in other regions
T3PKS	29	Alkylresorcinol (100%)	29	Alkylresorcinol (100%)	29	Alkylresorcinol (100%)			Conserved, ATCC 10595 -missing	8	T3PKS	Alkylresorcinol (100%)	Conserved in other regions
Terpene,NRPS	30	N.M.	30	N.M.	30	A54145 (3%)	1	A54145 (3%)	Conserved				Missing
										28	NRPS, T1PKS, Lanthipeptide	Oxazolomycin (21%)	15439-specific
										29	NRPS, T1PKS	Streptolydigin (21%)	15439-specific
										30	Betalactone, NRPK-like	kanamycin (4%)	15439-specific
										31	Terpene	N.M.	15439-specific

Table S7. Protein sequence variations of chloramphenicol biosynthetic genes among the three *S. venezuelae* strains.

Gene	Function	Position	10712	21113	10595	Domain_spcific hits
cmlR	Transcriptional activator	234	Gly	–	Gly	–
		235	Pro	–	Pro	–
		326	Ser	Ala	Ser	–
		332	Ala	Ala	Val	–
		336	Glu	Glu	Asp	–
cmlL	Phosphopantetheinyl transferase	14	Ala	Ala	Thr	–
		226	Ala	Thr	Thr	–
		230	Ala	Val	Ala	–
		234	Ala	Ala	Thr	–
cmlN	Integral membrane ion antiporter	1	Val	–	–	–
		2	Phe	–	–	–
		59	Ala	Ala	Val	Na_H_Exchanger
		221	Thr	Ala	Ala	–
		233	His	Arg	His	–
		235	Arg	Arg	Gln	–
		237	Val	Val	Ala	–
		238	Ala	Gly	Ala	–
		244	Val	Val	Met	–
		248	Gly	Glu	Gly	–
		254	–	–	Arg	–
		255	–	–	Val	–
		256	–	–	Gln	–
		257	–	–	Gly	–
		258	–	–	Val	–
		259	–	–	Ala	–
		260	–	–	Gly	–
		261	–	–	Arg	–
		262	–	–	Val	–
		263	–	–	Gln	–
		264	–	–	Gly	–
		265	–	–	Val	–
		266	–	–	Val	–
		267	–	–	Gly	–
		270	Gln	Lys	Lys	–
		272	Thr	Thr	Pro	–
		388	Ala	Val	Ala	Na_H_Exchanger
		389	Ala	Ala	Thr	Na_H_Exchanger
cmlF	Chloramphenicol efflux pump	186	Ser	Ala	Ala	MFS_1
cmlE	DAHP synthase	–				
cmlD	4-amino-4-deoxychorismate mutase	–				
cmlC	4-amino-4-deoxyprephenate dehydrogenase	6	Arg	Arg	Gln	–
		254	Ile	Val	Val	–
		260	Arg	–	Arg	–
		261	Ala	–	Val	–
		262	Glu	–	Asp	–
		263	Asp	–	Asp	–
		264	Pro	–	Pro	–
		265	Asp	–	Asp	–
		267	Ala	Val	Val	–
		269	Asp	Ala	Asp	–
		271	Asp	Gly	Gly	–

		273	Thr	Ala	Ala	–
		275	Asn	Ala	Val	–
		282	Cys	Cys	Phe	–
		293	Glu	Gly	Glu	–
cmlB	4-amino-4-deoxychorismate synthase	42	Leu	Val	Val	GATase
		245	Phe	Tyr	Phe	Anth_synt_I_N
		286	Ser	Asn	Ser	Anth_synt_I_N
		302	Asp	Glu	Glu	Anth_synt_I_N
		386	Val	Ala	Ala	–
		681	Ala	Val	Ala	–
cmlA	Nonheme iron monooxygenase	116	Val	Ala	Val	CmlA_N
		123	Ala	Pro	Ala	CmlA_N
		200	Ala	Thr	Thr	CmlA_N
		209	Thr	Ala	Ala	CmlA_N
		213	Asp	Gln	Asp	CmlA_N
		216	Ala	Glu	Ala	CmlA_N
		235	Thr	Thr	Ala	CmlA_N
		242	Pro	Pro	Ser	–
		245	Ala	Ala	Asp	–
		247	–	Gly	Gly	–
		248	–	Ser	Ser	–
		249	–	Ala	Ala	–
		250	–	Ser	Ser	–
		251	–	Gly	–	–
		252	–	Ser	–	–
		253	Ser	Ser	Ala	–
		255	Glu	Glu	Asp	UlaG
cmlP	Nonribosomal peptide synthase	1	–	Val	Val	–
		120	Ala	Ser	Ala	AMP-binding
		135	Ala	Glu	Glu	AMP-binding
		190	Gln	Gln	Glu	AMP-binding
		304	Ser	Thr	Thr	AMP-binding
		456	Leu	Val	Val	–
		582	Arg	Arg	His	–
		586	Thr	Ala	Thr	–
		665	Ala	Ala	Ser	Epimerase
		743	Asp	Asp	Ser	Epimerase
		852	Arg	Arg	Pro	–
		853	Thr	Ala	Ala	–
		944	Glu	Gly	Gly	–
		946	Thr	Thr	Pro	–
cmlH	Amidase	1	Met	Val	Val	–
		17	Arg	Gly	Gly	–
		96	Gly	Ser	Gly	–
		161	Asp	Asp	Glu	–
cmlI	N-oxygenase	182	Glu	Ala	Glu	AurF
cmlM	Putative acyl carrier protein	–				–
cmlJ	Short-chain dehydrogenase	64	Ala	Ala	Glu	adh_short_C2
		216	Arg	Arg	Pro	adh_short_C2
cmlK	Acyl-CoA-ACP synthetase, AMP-ligase	19	Tyr	Phe	Phe	AMP-binding
		119	Ala	–	Ala	AMP-binding
		120	Ala	–	Gly	AMP-binding
		121	Arg	–	Arg	AMP-binding
		122	Ala	–	Ala	AMP-binding

		123	Glu	–	Glu	AMP-binding
		124	Pro	–	Pro	AMP-binding
		125	Ala	–	Ala	AMP-binding
		126	Gly	–	Gly	AMP-binding
		127	Pro	–	Pro	AMP-binding
		128	Ala	–	Ala	AMP-binding
		129	Gln	–	Gln	AMP-binding
		130	Leu	–	Leu	AMP-binding
		133	Ala	Val	Ala	AMP-binding
		135	Glu	Glu	Asp	AMP-binding
		137	Thr	Thr	Ala	AMP-binding
		138	Asp	Asp	Gln	AMP-binding
		369	His	Asp	Asp	AMP-binding
cmlS	Flavin-dependent halogenase	148	Arg	Gln	Arg	Trp_halogenase
		394	Ala	Ala	Val	Trp_halogenase
cmlT	Aldo-keto reductase	92	Pro	Pro	His	Aldo_ket_red
		252	Ala	Thr	Ala	Aldo_ket_red
		292	Ala	Thr	Ala	Aldo_ket_red
		299	Gly	Glu	Glu	Aldo_ket_red

Table S8. Protein sequence variations of jadomycin biosynthetic genes among the three *S. venezuelae* strains.

Gene	Function	Position	10712	21113	10595	Domain_spcific hits
jadR3	γ-butyrolactone-sensing activator/repressor	226	Ser	Thr	Gly	–
jadW1	γ-butyrolacton (SVB1) synthase	237	Ala	Val	Val	AfsA
		269	Ser	Ser	Thr	AfsA
		274	Asp	Glu	Asp	AfsA
		275	Pro	Ala	Pro	AfsA
		280	Ile	Val	Val	AfsA
		289	Ser	Ser	Thr	AfsA
jadW2	γ-butyrolacton (SVB1) synthase	14	Ile	Ile	Val	3Beta_HSD
		42	Pro	Leu	Pro	3Beta_HSD
		45	Ala	Ala	Pro	3Beta_HSD
		46	Ala	Thr	Ala	3Beta_HSD
		100	Asp	Ala	Asp	3Beta_HSD
		108	Thr	Thr	Ser	3Beta_HSD
		259	Arg	Gly	Gly	–
		306	Ala	Ala	Pro	–
jadW3	γ-butyrolacton (SVB1) synthase	28	Thr	Ala	Ala	adh_short_C2
jadR2	pseudo γ-butyrolacton receptor	–				–
jadR1	Atypical response regulator	–				–
jadJ	Acetyl-coenzyme A carboxylase	248	Glu	Pro	Ala	CPSase_L_D2
		357	Pro	Ala	Ala	Biotin_carb_C
		424	Thr	Thr	Arg	Biotin_carb_C
jadI	Cyclase	–				–
jadA	Ketoacyl synthase	–				–
jadB	Chain length factor	43	Thr	Ser	Ser	ketoacyl-synt
		319	Thr	Ala	Thr	Ketoacyl-synt_C
jadC	Acyl carrier protein	–				–
jadE	Ketoreductase	–				–
jadD	Cyclase/Dehydratase	–				–
jadF	Oxygenase/Dehydratase	19	Asp	–	Asp	–
		20	Thr	–	Thr	–
		21	Pro	–	Pro	–
		107	Pro	Ser	Pro	FAD_binding_3
		108	Gly	Ser	Ser	FAD_binding_3
		163	Asp	Ala	Asp	FAD_binding_3
		193	Glu	Glu	Gly	FAD_binding_3
		267	Gly	Ala	Gly	FAD_binding_3
		451	Ala	Thr	Thr	–
		452	–	Gly	Gly	–
		453	–	–	Ala	–
jadG	Anthrone oxygenase	89	Gln	Gln	Glu	–
jadH	Oxygenase/Dehydratase	1	–	Met	Met	–
		2	–	Ser	Ser	–
		3	–	Ala	Ala	–
		4	–	Val	Val	–
		5	–	Thr	Thr	–
		6	–	Thr	Thr	–
		7	–	Thr	Thr	–
		8	–	Pro	Pro	–
		9	–	Glu	Glu	–
		10	–	Ala	Ala	–
		11	–	Gly	Gly	–
		12	–	Phe	Phe	–
		13	–	Asp	Asp	–
		14	–	Ala	Ala	FAD_binding_3
		15	–	Asp	Asp	FAD_binding_3
		16	–	Val	Val	FAD_binding_3

		17	–	Ile	Ile	FAD_binding_3
		18	–	Val	Val	FAD_binding_3
		19	–	Val	Val	FAD_binding_3
		20	–	Gly	Gly	FAD_binding_3
		21	–	Ala	Ala	FAD_binding_3
		22	–	Gly	Gly	FAD_binding_3
		23	–	Pro	Pro	FAD_binding_3
		24	–	Thr	Thr	FAD_binding_3
		25	–	Gly	Gly	FAD_binding_3
		26	–	Leu	Leu	FAD_binding_3
		394	Asp	Asp	Glu	–
		459	Thr	Thr	Ala	–
		466	Thr	Ser	Thr	–
		501	Ala	Thr	Ala	–
		507	Leu	Arg	Leu	–
		527	Ala	Thr	Ala	–
jadK	Hydrolase	101	His	His	Asp	Abhydrolase_6
		121	Thr	Ala	Ala	Abhydrolase_6
jadL	Efflux pump	66	Leu	Val	Leu	MFS_3
		86	Ala	Val	Val	MFS_3
		129	Thr	Thr	Ala	MFS_3
		207	Gly	Glu	Glu	MFS_3
		215	Ala	Ala	Val	MFS_3
		218	Glu	Lys	Lys	MFS_3
		222	Asp	Glu	Glu	MFS_3
		316	Ala	Thr	Thr	MFS_3
		426	Pro	Leu	Leu	–
		429	Gly	Asp	–	–
		430	Pro	Pro	–	–
		431	Asp	Asp	–	–
		432	Ser	Ala	–	–
		433	Asp	Asp	–	–
		434	Ser	Ser	–	–
		435	Pro	Pro	–	–
		441	Pro	Ser	Ser	–
jadM	Phosphopantetheinyl transferase	21	Pro	Pro	Thr	–
		28	Ala	Thr	Ala	–
		37	Ala	Val	Ala	–
		148	Glu	Glu	Asp	ACPS
		194	Ala	Ala	Val	–
		213	Leu	Val	Leu	–
		214	His	Arg	Arg	–
		234	–	Thr	Thr	–
		237	His	His	Asp	–
		243	Gln	His	His	–
jadN	Acyl-CoA carboxylase subunit beta	237	Val	Val	Ala	Carboxyl_trans
		245	Leu	Leu	Ile	Carboxyl_trans
jadN2	Acyl-CoA carboxylase subunit epsilon	1	–	Met	–	–
		2	–	Thr	–	–
		3	–	Thr	–	–
		4	–	Glu	–	–
		5	–	Ala	–	–
		6	–	arg	–	–
		7	–	Leu	–	–
		8	–	Pro	–	–
		9	–	Gly	–	–
		10	–	Thr	–	–
		11	–	Asp	–	–
		12	–	arg	–	–
		13	Met	Leu	Met	ACC_epsilon

		46	Thr	Ala	Ala	ACC_epsilon
jadX	Nuclear transport factor 2 (NTF2)-like protein	18	Ala	Ala	Thr	–
		36	Ala	Glu	Glu	SnoaL_4
		141	His	Gln	His	SnoaL_4
		146	Pro	Ser	Pro	SnoaL_4
jadO	NDP-hexose-2,3-dehydratase	8	Pro	Ala	Ala	–
		209	Gly	Val	Gly	Hexose_dehydrat
		379	Thr	Ser	Ser	Hexose_dehydrat
		392	Thr	Ala	Ala	Hexose_dehydrat
jadP	NDP-hexose-3-ketoreductase	1	–	Met	Met	–
		2	–	Thr	Thr	–
		3	–	Ser	Ser	–
		4	–	Thr	Thr	–
		5	–	Pro	Pro	–
		6	–	Ala	Ala	–
		7	–	Pro	Pro	–
		8	Met	Leu	Leu	GFO_IDH_MocA
		48	Val	Ala	Ala	GFO_IDH_MocA
		152	Ala	Ser	Ala	–
		172	Gly	Arg	Gly	–
		278	Leu	Ile	Leu	–
		304	His	His	Arg	–
jadQ	NDP-glucose synthase	82	Gln	Gln	Glu	NTP_transferase
		95	Asp	Asp	Glu	NTP_transferase
jadS	Glycosyltransferase	390	Asn	Asp	Asp	–
jadT	NDP-hexose-4,6-dehydratase	–				–
jadU	NDP-hexose-5-epimerase	1	–	Met	–	–
		2	–	Thr	–	–
		3	–	Thr	–	–
		4	–	Ser	–	–
		34	Tyr	Phe	Tyr	dTDP_sugar_isom
		60	Arg	Lys	Arg	dTDP_sugar_isom
		118	Val	Ile	Val	dTDP_sugar_isom
		185	Asp	Ala	Asp	–
jadV	NDP-hexose-4-ketoreductase	66	Val	Val	Ala	Epimerase
*jaR**	TetR-family regulator	–				–
jadY	Unknown	36	Ala	Ala	Thr	FMN_red
		57	Ser	Ser	Thr	FMN_red

Reference

1. El-Gebali, S.; Mistry, J.; Bateman, A.; Eddy, S.R.; Luciani, A.; Potter, S.C.; Qureshi, M.; Richardson, L.J.; Salazar, G.A.; Smart, A., et al. The Pfam protein families database in 2019. *Nucleic Acids Research* **2019**, *47*, D427-D432.
2. Thanapipatsiri, A.; Gomez-Escribano, J.P.; Song, L.J.; Bibb, M.J.; Al-Bassam, M.; Chandra, G.; Thamchaipenet, A.; Challis, G.L.; Bibb, M.J. Discovery of unusual biaryl polyketides by activation of a silent *Streptomyces venezuelae* biosynthetic gene cluster. *Chembiochem* **2016**, *17*, 2189-2198.
3. Inahashi, Y.; Zhou, S.S.; Bibb, M.J.; Song, L.J.; Al-Bassam, M.M.; Bibb, M.J.; Challis, G.L. Watasemycin biosynthesis in *Streptomyces venezuelae*: thiazoline C-methylation by a type B radical-SAM methylase homologue. *Chem Sci* **2017**, *8*, 2823-2831.
4. Rabe, P.; Rinkel, J.; Klapschinski, T.A.; Barra, L.; Dickschat, J.S. A method for investigating the stereochemical course of terpene cyclisations. *Org Biomol Chem* **2016**, *14*, 158-164.
5. Goto, Y.; Li, B.; Claesen, J.; Shi, Y.X.; Bibb, M.J.; van der Donk, W.A. Discovery of unique lanthionine synthetases reveals new mechanistic and evolutionary insights. *Plos Biol* **2010**, *8*.
6. Ehrlich, J.; Gottlieb, D.; Burkholder, P.R.; Anderson, L.E.; Pridham, T.G. *Streptomyces venezuelae*, n. sp., the source of chloromycetin. *J Bacteriol* **1948**, *56*, 467-477.
7. Sidda, J.D.; Song, L.J.; Poon, V.; Al-Bassam, M.; Lazos, O.; Buttner, M.J.; Challis, G.L.; Corre, C. Discovery of a family of gamma-aminobutyrate ureas via rational derepression of a silent bacterial gene cluster. *Chem Sci* **2014**, *5*, 86-89.
8. Doull, J.L.; Ayer, S.W.; Singh, A.K.; Thibault, P. Production of a novel polyketide antibiotic, jadomycin B, by *Streptomyces venezuelae* following heat shock. *J Antibiot (Tokyo)* **1993**, *46*, 869-871.
9. Kodani, S.; Komaki, H.; Suzuki, M.; Kobayakawa, F.; Hemmi, H. Structure determination of a siderophore peucechelin from *Streptomyces peucetius*. *Biometals* **2015**, *28*, 791-801.
10. Som, N.F.; Heine, D.; Holmes, N.A.; Munnoch, J.T.; Chandra, G.; Seipke, R.F.; Hoskisson, P.A.; Wilkinson, B.; Hutchings, M.I. The conserved actinobacterial two-component system MtrAB coordinates chloramphenicol production with sporulation in *Streptomyces venezuelae* NRRL B-65442. *Front Microbiol* **2017**, *8*, 1145.
11. Carter, H.E.; Gottlieb, D.; Anderson, H.W. Chloromycetin and Streptothricin. *Science* **1948**, *107*, 113.
12. Xue, Y.; Zhao, L.; Liu, H.W.; Sherman, D.H. A gene cluster for macrolide antibiotic biosynthesis in *Streptomyces venezuelae*: architecture of metabolic diversity. *Proc Natl Acad Sci U S A* **1998**, *95*, 12111-12116.
